# Identifying cell-enriched miRNAs in kidney injury and repair

**DOI:** 10.1172/jci.insight.140399

**Published:** 2020-12-17

**Authors:** Katie L. Connor, Oliver Teenan, Carolynn Cairns, Victoria Banwell, Rachel A.B. Thomas, Julie Rodor, Sarah Finnie, Riinu Pius, Gillian M. Tannahill, Vishal Sahni, Caroline O.S. Savage, Jeremy Hughes, Ewen M. Harrison, Robert B. Henderson, Lorna P. Marson, Bryan R. Conway, Stephen J. Wigmore, Laura Denby

**Affiliations:** 1 Centre for Cardiovascular Science, University of Edinburgh, Edinburgh, United Kingdom.; 2 Edinburgh Transplant Unit, Edinburgh Royal Infirmary, Edinburgh, United Kingdom.; 3 Centre for Inflammation Research and; 4 Centre for Medical Informatics, Usher Institute, University of Edinburgh, Edinburgh, United Kingdom.; 5 Medicines Research Centre, GlaxoSmithKline, Stevenage, United Kingdom.

**Keywords:** Nephrology, Bioinformatics, Noncoding RNAs, Organ transplantation

## Abstract

Small noncoding RNAs, miRNAs (miRNAs), are emerging as important modulators in the pathogenesis of kidney disease, with potential as biomarkers of kidney disease onset, progression, or therapeutic efficacy. Bulk tissue small RNA-sequencing (sRNA-Seq) and microarrays are widely used to identify dysregulated miRNA expression but are limited by the lack of precision regarding the cellular origin of the miRNA. In this study, we performed cell-specific sRNA-Seq on tubular cells, endothelial cells, PDGFR-β^+^ cells, and macrophages isolated from injured and repairing kidneys in the murine reversible unilateral ureteric obstruction model. We devised an unbiased bioinformatics pipeline to define the miRNA enrichment within these cell populations, constructing a miRNA catalog of injury and repair. Our analysis revealed that a significant proportion of cell-specific miRNAs in healthy animals were no longer specific following injury. We then applied this knowledge of the relative cell specificity of miRNAs to deconvolute bulk miRNA expression profiles in the renal cortex in murine models and human kidney disease. Finally, we used our data-driven approach to rationally select macrophage-enriched miR-16-5p and miR-18a-5p and demonstrate that they are promising urinary biomarkers of acute kidney injury in renal transplant recipients.

## Graphical abstract

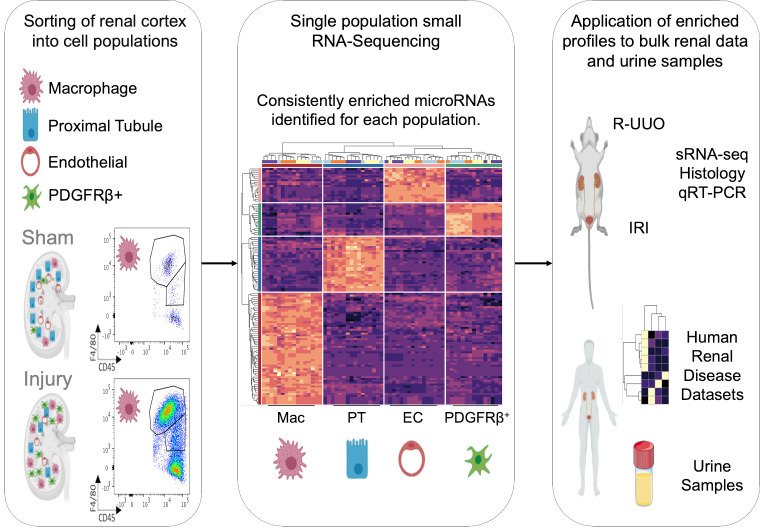

## Introduction

Small noncoding microRNAs (miRNAs) are important both as key pathogenic regulators (1, 2) and as biomarkers of renal disease (3). Their capacity to drive renal disease has now been recognized and begun to be translated from preclinical studies (4, 5) to clinical trials of miRNA inhibitor–based (anti-miR) therapies in renal disease (6). The ability of miRNAs to be stably detected in samples, including urine and serum, makes them attractive biomarkers, offering the potential to provide a real-time reflection of the molecular and pathological landscape of the kidney, without the need for renal biopsy.

With the advent of microarray and later small RNA-sequencing (sRNA-seq), the bulk tissue transcriptome-wide profile of miRNAs has been characterized across various renal conditions (7–12). However, the expression of a given miRNA in such whole-tissue transcriptomics reflects both the abundance of that miRNA in each cell type and the proportion of that cell type within the sample (13). This is problematic both for disease process and for biomarker research, as the proportion of each renal cell type may vary considerably among renal health, injury, and repair. For example, it is evident that inflammatory renal disease induces a large increase in the ratio of inflammatory cells to tubular cells as compared with that in a healthy kidney (14, 15).

Unlike analysis of gene expression (13), defining cell-specific miRNA expression within the kidney is hampered by the lack of equivalent single-cell sequencing methodology for miRNAs. Although “kidney-enriched” miRNAs, such as miR-192, miR-194, and the miR-30 family, have been identified by sRNA-Seq across multiple organs (16, 17), there are shortcomings to this approach. First, it is unable to detect differences between cell types in the kidney, essential for the unambiguous interpretation of a biopsy, for example. Second, it rests on the assumption that cell enrichment remains stable across injury and repair. SRNA-Seq to detect the differentially expressed miRNAs (DEmiRNAs) in microdissected proximal tubules and glomeruli has provided information on the cell-specific miRNA changes in diabetic nephropathy, IgA nephropathy, and focal segmental glomerulosclerosis (FSGS) (18). Although the most highly expressed miRNAs are listed in both proximal tubules and glomeruli, this does not equate to cell selectivity of these miRNAs. Moreover, the DEmiRNAs described, while undoubtedly important, may be masked when examining a whole-tissue biopsy sample.

Understanding the cell-specific miRNA expression in the kidney is essential, both to contextualize whole-tissue miRNA changes and to identify miRNAs that may have potential utility as either therapeutic targets or biomarkers. Because only the most abundant miRNAs in a cell are thought to mediate mRNA target suppression (19), profiling the cellular miRNA expression may guide researchers as to which miRNAs to mechanistically interrogate and in which cell type. Similarly, this knowledge may enhance the rational selection of biomarkers in kidney disease, based on the expected cellular pathology of the condition.

To address this knowledge gap, we performed sRNA-Seq on discrete renal cell populations obtained from mouse kidney in health, following injury, and during repair. The aim of the study was to identify miRNAs that stringently remained enriched in specific cell types across acute injury, late injury, and repair in the kidney, which could be reasonably applied as cell-enriched markers in both acute kidney injury and chronic kidney disease. For our analysis pipeline, we combined an unbiased clustering approach and validated it with a supervised methodology, identifying cell-specific miRNAs in the kidney and creating an interactive platform for others to explore these results and contextualize their own findings. We then use this knowledge to deconvolute DEmiRNA expression in whole-tissue data obtained from a further mouse model of renal injury and in data sets from human renal disease. Finally, we apply this knowledge to rationally select miRNAs having potential as disease biomarkers, identifying macrophage-enriched miR-18a-5p and miR-16-5p as urinary biomarkers of acute kidney injury following transplantation.

## Results

### Reversible unilateral ureteric obstruction model induces kidney injury followed by resolution.

**Figure 1:**
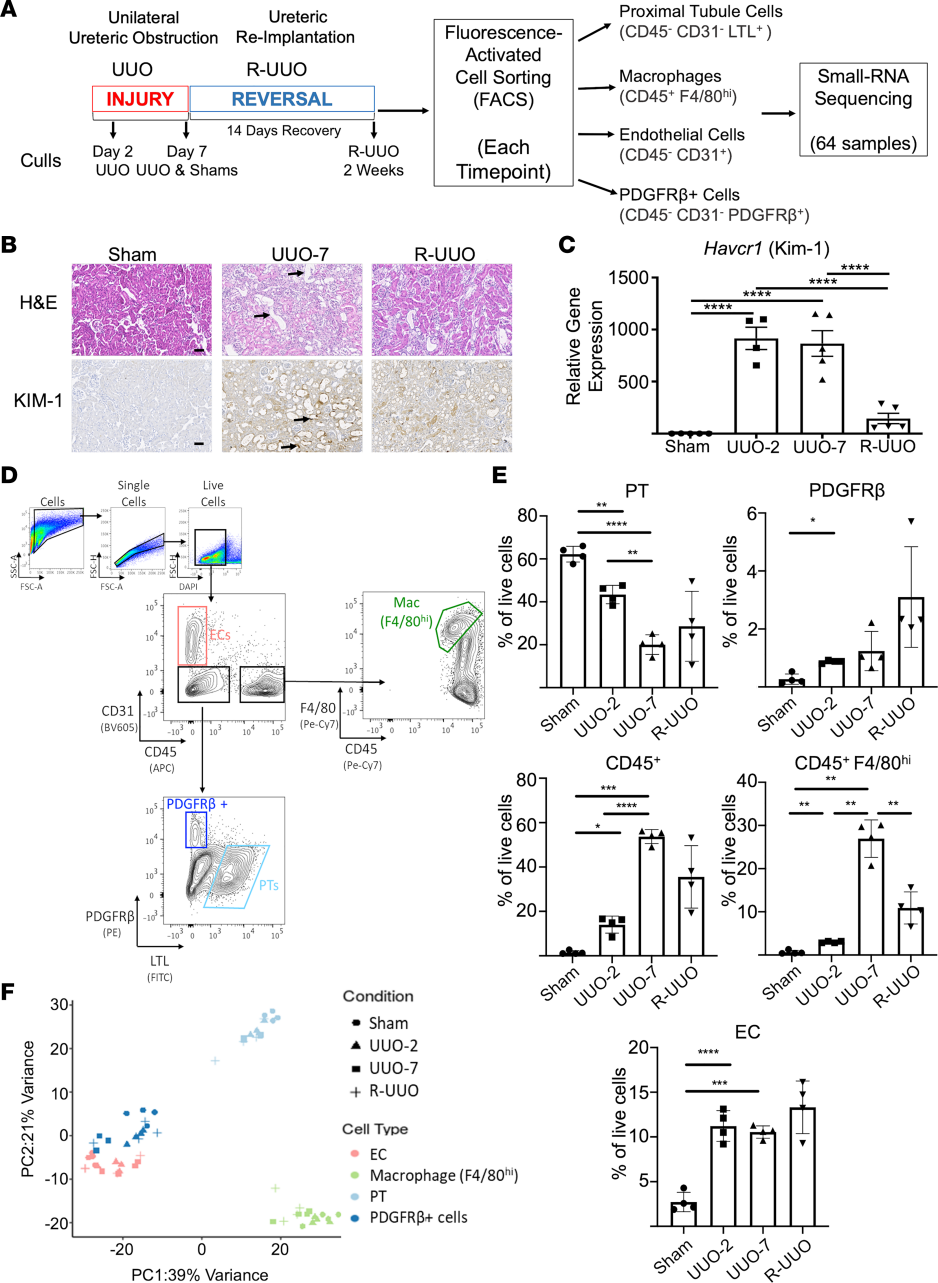
R-UUO model induces kidney injury followed by resolution. (**A**) Schematic of the experimental process. Injury was induced by 2 or 7 days of UUO. After 7 days of UUO, ureteric reimplantation facilitates reversal of obstruction. At each time point proximal tubular cells, macrophages, endothelial cells, and PDGFR-β^+^ cells were isolated by fluorescence-activated cell sorting (FACS) for small RNA-sequencing (sRNA-Seq). (**B** and **C**) By UUO-7 there were marked tubular dilatation (arrows), cellular infiltration and increased tubular expression of kidney injury marker-1 (KIM-1/Havcr1) (arrows). Upon reversal (R-UUO), there was a rapid decline in Havcr1 expression, indicating successful reversal of renal tubular injury. Scale bar: 50 μm. Data shown as mean ± SEM. One-way ANOVA with Tukey’s multiple-comparison test. ****P < 0.0001. (**D**) The renal cortex was digested into single cells, which were separated by FACS into endothelial cells (ECs) (CD45^–^CD31^+^), macrophages (CD45^+^F4/80^hi^), proximal tubular cells (PT cells) (CD45^–^CD31^–^LTL^+^), and PDGFR-β^+^ (CD45^–^CD31^–^PDGFR-β^+^) cells. (**E**) The proportions of these sorted cell populations varied with injury and repair (*n* = 4 per group). Data presented as mean ± SD analyzed by Welch’s ANOVA test with Dunnett’s multiple-comparison test. *P < 0.05, **P < 0.01, ***P < 0.001, ****P < 0.0001. (**F**) Principal component analysis of sRNA-Seq data demonstrates that cell type accounts for the key variance in miRNA expression (top 500 most variant miRNAs shown, 64 samples). UUO-2, UUO cull day 2; UUO-7, UUO cull day 7; R-UUO, cull following 2 weeks’ reversal of UUO.

The reversible unilateral ureteric obstruction (R-UUO) model (20–22) allowed us to map renal miRNA expression in normal, injured, and repairing kidney tissue (Figure 1A). One week following UUO, there was widespread renal tubular dilatation (Figure 1B) and a marked increase in tubular expression of kidney injury molecule-1 (KIM-1) encoded by the gene *Havcr1* (Figure 1, B and C). Following reimplantation of the ureter to reverse obstruction, there was a reduction in tubular dilatation and a significant decline in KIM-1 expression, with an 83% reduction in Havcr1 expression by 2 weeks of reversal of the injury (*P* < 0.0001 vs. UUO-7), indicating effective reversal of obstructive tubular injury (Figure 1, B and C).

The kidney is a complex organ with multiple cell types. In order to identify cell-selective miRNAs, we chose to examine the sRNA profile of 4 of the key cell types implicitly involved in kidney injury and repair. We used FACS of digested cortical kidney tissue to isolate endothelial cells (ECs; CD45^–^CD31^+^), F4/80^hi^ macrophages (Macs; CD45^+^F4/80^hi^), PDGFR-β^+^ stromal cells/fibroblasts (CD45^–^CD31^–^), and proximal tubular cells (PT cells; CD45^–^CD31^–^LTL^+^) (Figure 1D). During injury (UUO), the proportion of live PT cells significantly decreased, whereas the proportion of CD45^+^ immune cells, macrophages, and PDGFR-β^+^ cells significantly increased (Figure 1E). Upon reversal, there was significant reduction in the proportion of macrophages but a persistence of CD45^+^ cells, which likely reflects T cell presence during the repair phase as has been observed before (22, 23). An increase in ECs with injury was noted; this may be a consequence of increased expression of CD31 upon endothelial activation and/or increased liberation of ECs from injured tissue. To assess the purity of our populations, we demonstrated that marker gene expression was specific to the corresponding cell population (Supplemental Figure 1A; supplemental material available online with this article; https://doi.org/10.1172/jci.insight.140399DS1).

We performed sRNA-Seq on each of the isolated cell types from sham-operated animals, at 2 and 7 days after UUO (UUO-2, UUO-7) and at 2 weeks following R-UUO (*n* = 4 per group). Small RNAs were annotated to miRBase and miRNA expression was analyzed. Following quality control (Supplemental Figure 1B), our initial unbiased clustering demonstrated that the key variance within the miRNA expression data was due to cell type rather than the experimental condition, with the least variance observed between the endothelial and PDGFR-β^+^ cells (Figure 1F).

### Identification of cell-selective, consistently enriched miRNAs in the mouse kidney.

**Figure 2:**
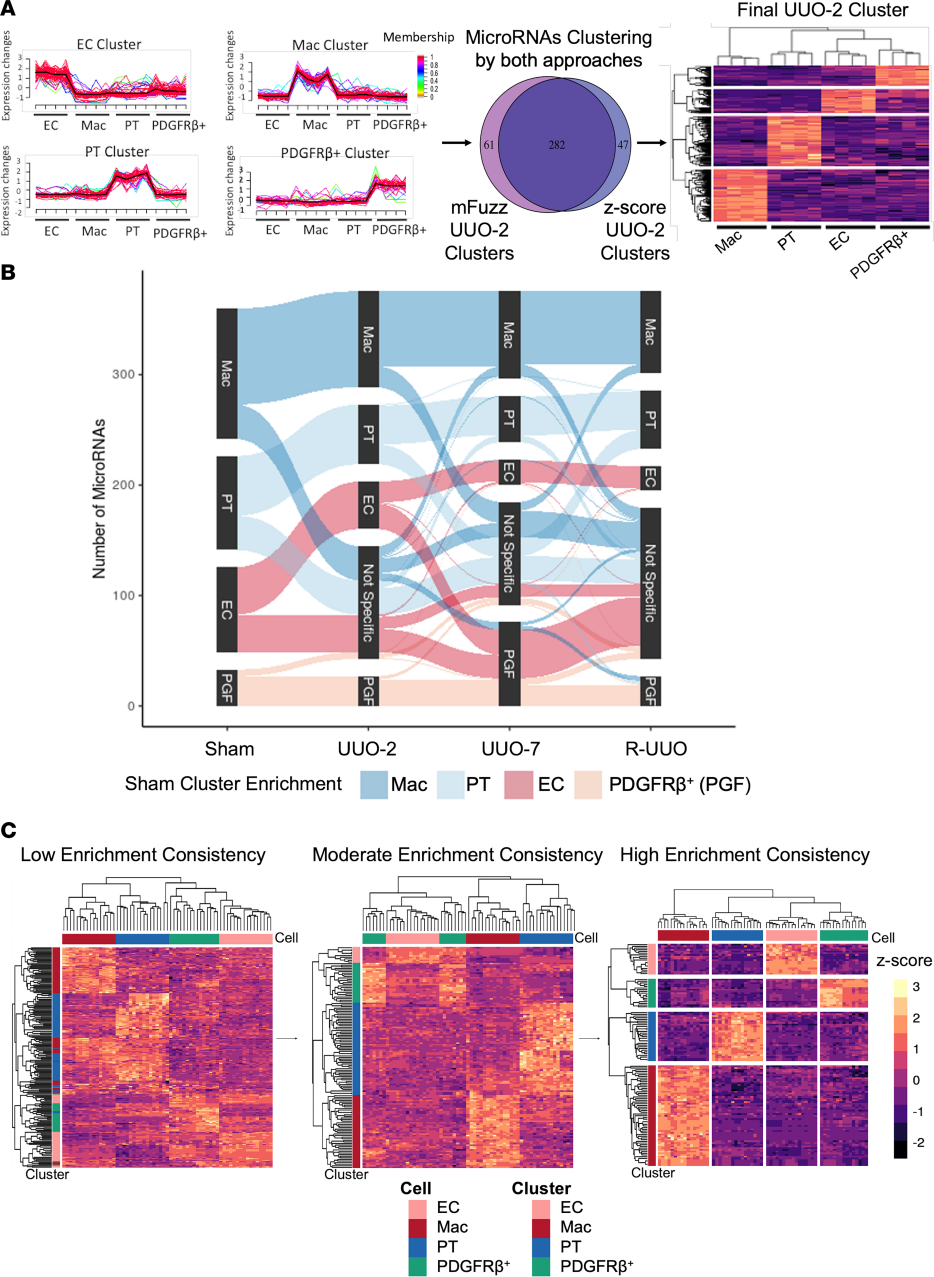
Development of kidney cell enrichment clusters. Analysis of single population sRNA-Seq of macrophage (Mac; *n* = 16), endothelial (EC; *n* = 16), PDGFR-β^+^ (*n* = 15), and proximal tubular (PT; *n* = 16) sorted cells (*n* = 3–4 per time point) from the R-UUO model to identify miRNAs specific for each cell type. (**A**) For each time point, cell clusters were developed using a combination of unbiased fuzzy clustering (mFuzz) and filtering relative expression (*z* score) in each cell type (abbreviated example shown of UUO-2, full data Supplemental Figures 2–5). (**B**) Sankey plot showing the trajectory of each individual miRNA enriched at baseline (Sham). Many miRNAs either became non–cell specific or occasionally switched cell type enrichment during renal injury and repair. (**C**) On unsupervised clustering, miRNAs in high enrichment consistency clusters were noted to have greater cell specificity when mapped to all time points. Heatmap: Each row represents a miRNA; each column is a sample, with clustering by Euclidian distances. UUO, unilateral ureteric obstruction; R-UUO, reversal of UUO (2 weeks’ reversal); PGF/PDGFR-β, platelet-derived growth factor receptor–β.

With this knowledge, we then identified cell type–specific miRNA clusters in the different experimental conditions (Figure 2A and Supplemental Figures 2–5). First, we performed unsupervised, unbiased fuzzy clustering of data from each time point using mFuzz (ref. 24, Figure 2A, and Supplemental Figures 2–5). Because we knew the cellular origin of our samples a priori, we were able to combine this unsupervised approach with filtering by the relative expression for each miRNA within each cell type, enhancing the stringency of our selected enriched miRNAs. We defined those miRNAs with relatively higher expression (mean cell type *z* score > 1.15) as enriched (Supplemental Figure 6). The 2 approaches demonstrated significant overlap and, taking forward those that replicated across both approaches, we identified miRNAs enriched in each cell type for each experimental condition: sham (*n* = 313), UUO-2 (*n* = 282), UUO-7 (*n* = 330), and R-UUO (*n* = 232) (Table 1 and Supplemental Figure 6).

Organ atlas studies to date have primarily been performed on healthy animals, with the assumption that reported cell-specific miRNAs will remain enriched, despite transcriptional changes occurring during injury and repair, and thus represent consistent cell markers (25). We demonstrate that across the cell types, only 129 out of 408 (31.6%) miRNAs maintain consistent cell-specific enrichment at all time points (Table 1). On profiling the trajectory of sham-enriched miRNAs, it could be observed that with injury and repair a significant proportion either was no longer cell specific or became enriched in another cell type completely (Figure 2B). Importantly, this may confound the interpretation of their expression in whole-tissue RNA data sets of uninjured tissue.

**Figure 3:**
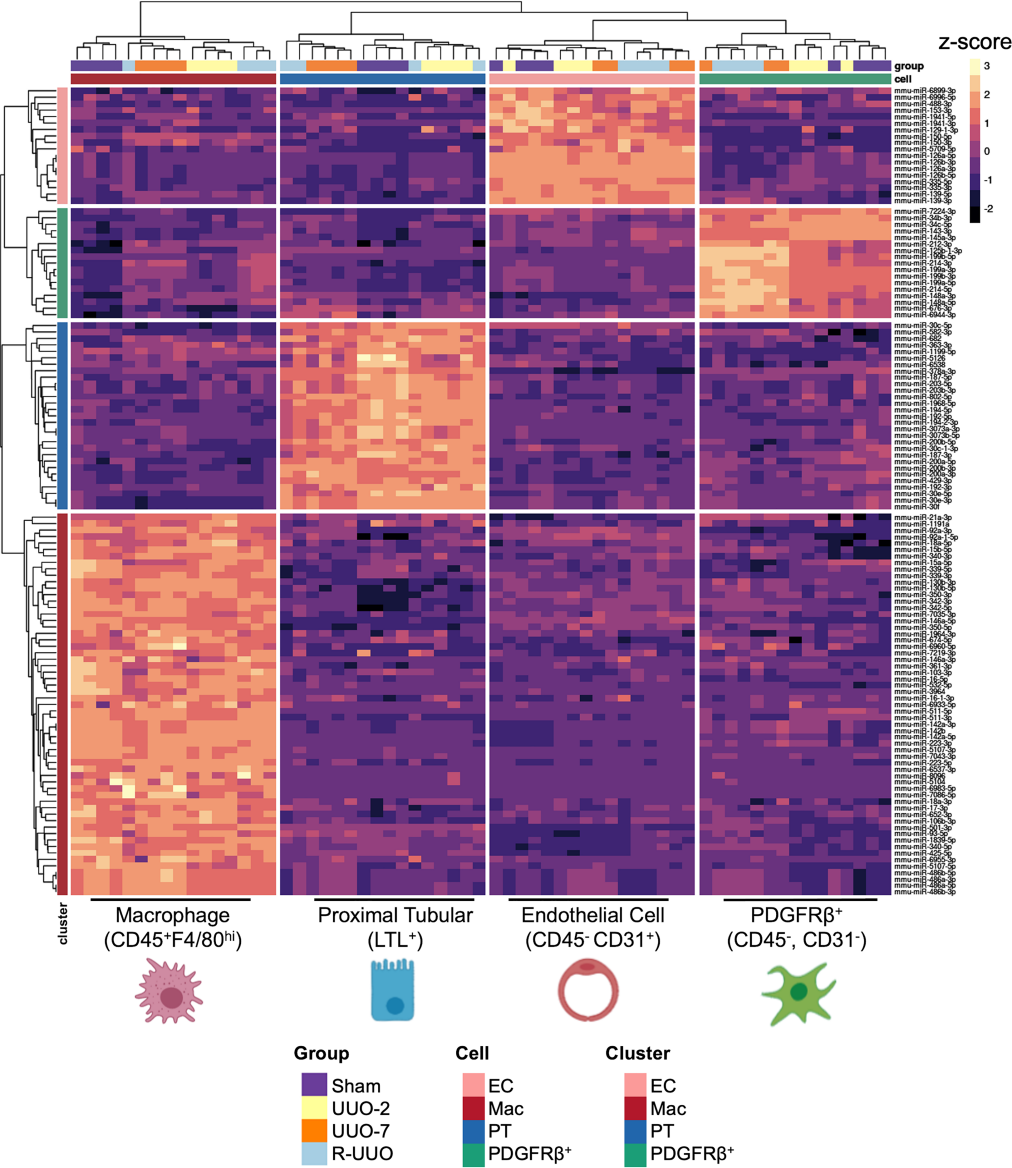
MiRNAs with consistent specific cell enrichment. MiRNAs demonstrating consistent cell type enrichment across all 4 experimental conditions are presented (*n* = 129). Each column represents a sample, each row a miRNA, and the color the relative expression (*z* score). Heatmap produced using unsupervised clustering by Euclidian distances. This data set can be fully explored at http://www.kidney-enriched-micrornas.com/ UUO, unilateral ureteric obstruction; R-UUO, reversal of UUO; Cell, cell origin from FACS; Cluster, assigned enrichment group.

On unsupervised reclustering of the data, it was evident that miRNAs exhibiting consistent cell-specific enrichment throughout all 4 experimental conditions had superior matching to their corresponding origin cell type (Figure 2C). As such, we defined miRNAs enriched in only 1 cell type and at 1, 2–3, and all 4 time points as having low (*n* = 134), moderate (*n* = 145), and high (*n* = 129) enrichment consistency, respectively. These highly enriched miRNAs (Figure 3), which appeared to be most consistently specific for each cell type, were taken forward for downstream analysis and deconvolution of bulk samples. The enrichment and dynamic changes of all profiled miRNAs can be explored in our interactive online platform available at http://www.kidney-enriched-micrornas.com

### Deconvolution of whole-kidney tissue sRNA-Seq data.

The R-UUO model exhibits increased inflammatory cell numbers and a loss of healthy proximal tubules with injury, with partial recovery in R-UUO (Figure 1, B and E), as we have previously described (22). We set out to explore whether the expression changes in whole-tissue sRNA-Seq of our highly enriched miRNAs mirrored the changes in the proportions of renal cells determined by flow cytometry. This would allow us to better contextualize changes in whole-kidney miRNA expression, applicable to a typical renal biopsy.

**Figure 4:**
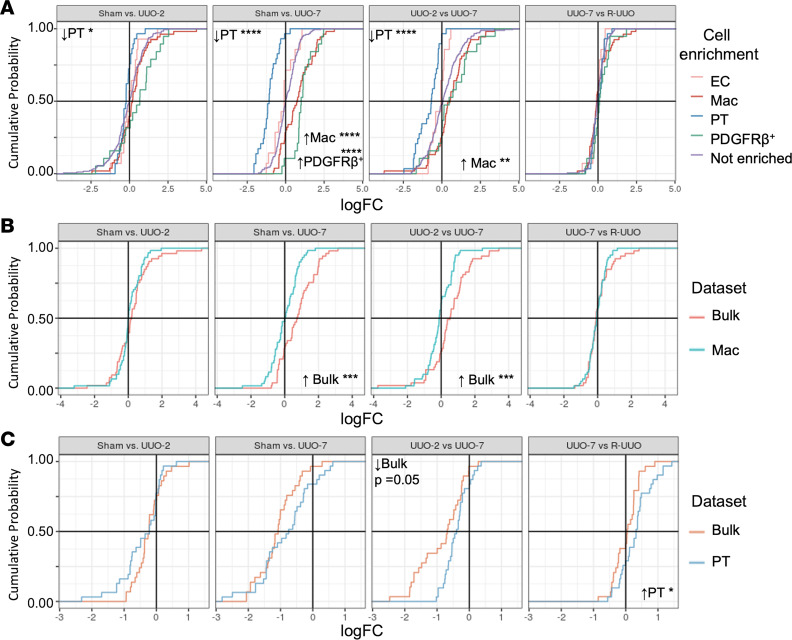
Global expression changes of cell-enriched miRNAs in the bulk and single-population data sets with injury and repair. (**A**) The cumulative distribution of the highly enriched miRNA expression changes (log fold change, LogFC) in the kidney tissue sRNA-Seq data set is shown. There is an increase in macrophage-enriched (Mac, green line) and PDGFR-β^+^ (red line) and decrease in proximal tubular cell–enriched (PT, blue line) miRNAs’ expression versus nonenriched miRNAs (purple line) in comparisons against sham (sham vs. UUO day 7 and sham vs. R-UUO) (ECs in pink). This is in keeping with the histology. (**B**) When comparing the macrophage-enriched miRNA expression in the bulk versus single-population Mac sRNA-Seq data sets, there was an upregulation of Mac enriched miRNAs within the bulk data set at UUO day 7 (vs. sham and UUO day 2) but not within the cells themselves. This suggests that the bulk changes may be due to changes in cellular proportions in the sample. (**C**) For PT cell–enriched miRNAs, there was a loss of expression both within the PT cells and to a greater extent the bulk tissue at UUO day 7 (vs. UUO day 2 and R–UUO). Upon reversal, PT cell–enriched miRNA expression increased within PT cells to a relatively greater extent than was evident in the bulk. empirical cumulative distribution function plots for all cell types and comparisons are shown in Supplemental Figures 7–9. Kolmogorov-Smirnov test was used to compare the distribution of the LogFC. *P < 0.05, **P < 0.01, ***P < 0.001, ****P < 0.0001.

To interrogate this, we first plotted the change in expression between experimental time points of the highly enriched miRNAs in each cell type in the whole-tissue sRNA-Seq data from the R-UUO model and compared this with that of nonenriched miRNAs. No overall shift in the expression of nonenriched miRNAs was observed between any of the experimental conditions, with the proportion of upregulated and downregulated miRNAs evenly distributed across the central axes (purple line, Figure 4A and Supplemental Figure 7). With early injury (sham vs. UUO-2), there was a loss of PT-enriched miRNAs, which became more marked by UUO-7 (blue line). As the injury progressed (UUO-7), there was a significant increase in the macrophage-enriched miRNAs (green line) versus both sham and UUO-2. In addition, PDGFR-β^+^–enriched miRNAs were upregulated at UUO-7, when established fibrosis was evident (red line). There were no significant changes in enriched and nonenriched miRNA expression between UUO-7 and R-UUO. Volcano plots displaying the profile of enriched cells for all experimental data sets and comparisons are available to visualize at http://www.kidney-enriched-micrornas.com

The global expression profiles of our cell-enriched miRNAs mirrored the changes observed in cellular proportions on flow cytometry (Figure 1E); for example, with injury, we observed an increase in macrophages (CD45^+^F4/80^hi^) and PDGFR-β^+^ cells, alongside an early and sustained loss of PT cells.

Within each cell type we identified that several miRNAs exhibited dynamic miRNA expression with injury and repair (Table 2). The expression profile of each individual miRNA can be explored at http://www.kidney-enriched-micrornas.com We also observed dynamic expression in the enriched miRNA population (Figure 3). For example, within the individual cell populations we found that in the PDGFR-β^+^ cells during late injury, there was substantial loss of enriched miR-143 and -145 and a significant increase in miR-199 and -214 (Supplemental Figure 8). We and others have previously shown that miR-214 is involved in renal injury and fibrosis (1, 26–28).

We next wished to determine whether the changes in the expression of enriched miRNAs over time within the bulk sRNA-Seq are due to differential expression of those miRNAs within each cell type or reflect changes in the proportion of cell types at each time point. We directly compared the expression changes in the bulk (cortical) and isolated macrophage sRNA-Seq data sets. While the expression of macrophage-enriched miRNAs increased with injury in the bulk sRNA-Seq data (orange); no increase was observed within the macrophages themselves (blue) (Figure 4B and Supplemental Figure 9A). Thus, the observed changes in the enriched miRNAs in the bulk sRNA-Seq are likely a consequence of either an influx of monocytes transitioning to F4/80^hi^ macrophages or proliferation of resident macrophages skewing the sample cellular composition in the kidney.

In the PT cells, there was downregulation in the expression of PT-enriched miRNAs with injury in both the whole-tissue (orange) and to a lesser extent within PT cells (blue) (Figure 4C and Supplemental Figure 9B). Upon reversal, there was a relative upregulation of PT cell–enriched miRNAs within the proximal tubular cells that was not evident within the whole-tissue sample. This would suggest changes observed in PT cell–enriched miRNAs on the whole-tissue sample may be in part compositional but are also related to downregulation of miRNA expression within the proximal tubular cells due to dedifferentiation during injury. Contrastingly, within the PDGFR-β^+^ cells, there was a large transcriptional increase of PDGFR-β^+^–enriched miRNAs, outstripping the measurable signal within the whole-tissue data set (Supplemental Figure 10A). Endothelial enriched miRNAs remained relatively stable in ECs and within the whole-tissue data set (Supplemental Figure 10B).

Taken together, this suggested these cell-enriched miRNAs could be used as a window to understand the cellular landscape in the injured and repairing kidney.

**Figure 5:**
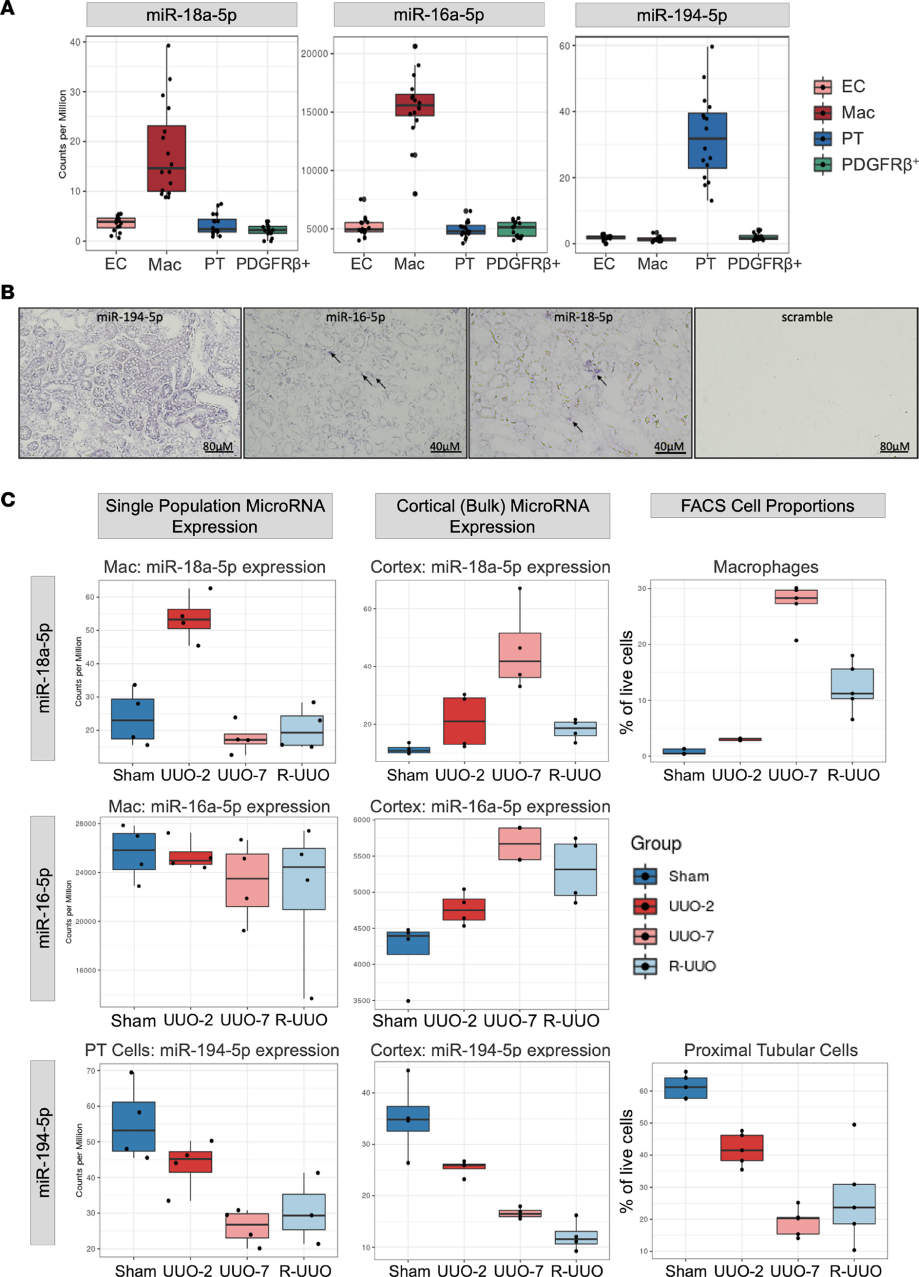
Selected enriched miRNA biomarkers’ expression and localization in the R-UUO model. (**A**) Expression of macrophage-enriched miR-18a-5p and miR-16-5p and proximal tubular cell–enriched miR-194-5p in the sorted cell populations from the R-UUO model demonstrating their enrichment for specific populations. (**B**) Localization of miRNA expression by in situ hybridization on R-UUO 3 μM FFPE sections using specific LNA double-labeled probes demonstrating a tubular pattern of expression for miR-194-5p and specific tubulointerstitial cellular expression for miR-16-5p and miR-18a-5p. (**C**) Comparisons of enriched miRNA expression with injury and repair in the matched single-population, renal cortex, and FACS cell proportions for macrophage-enriched miR-16-5p and miR-18a-5p and PT cell–enriched miR-194-5p. The cortical expression profile of the miRNAs mirrors the corresponding enriched cell type proportions of the sorted kidney. All data are expressed as the median ± 1.5 IQR.

MiRNAs are well conserved across phyla (29) and as such we next sought to assess whether the enriched miRNAs were also informative in murine and human acute kidney injury. We selected macrophage-enriched miR-18a-5p and miR-16-5p and PT cell–enriched miR-194-5p as they all demonstrated high cell-specific enrichment (Figure 5A). We confirmed their cellular localization in the R-UUO model by in situ hybridization, demonstrating that miR-194-5p was expressed predominantly in tubules while miR-16-5p and miR-18a-5p were expressed in the interstitium (Figure 5B). Two of these miRNAs, miR-18a-5p and miR-194-5p, also demonstrated early dynamic differential expression in response to injury and repair in the R-UUO model (Figure 5C) and correlated well with their corresponding cell number on flow cytometry (Figure 5C). MiR-16-5p was selected because it had stable expression regardless of injury, suggesting that the changes in miR-16-5p observed in bulk renal tissue may be more reflective of macrophage number (Figure 5C).

To gain insight into the potential biological pathways influenced by the dynamic expression of miR-18a-5p and miR-194-5p, we determined the validated targets (Supplemental Table 1. We then cross-referenced the list of validated targets with expression data from single-cell RNA-sequencing (scRNA-sequencing) performed in the same model (22) and looked for targets for which the gene expression was inversely correlated with miRNA expression. This analysis revealed that for macrophage-derived miR-18a-5p several targets were involved in the cell cycle, e.g., Ccnd1 (Supplemental Figure 11), and TGF-β signaling, e.g., Tgfbr1. Tgfbr1 signaling has been shown to be critical for functional specification in intestinal macrophages (30). For miR-194-5p targets included Runx1 (Supplemental Table 1), which has been shown to be upregulated in renal injury and to be targeted by 194-5p (31, 32).

**Figure 6:**
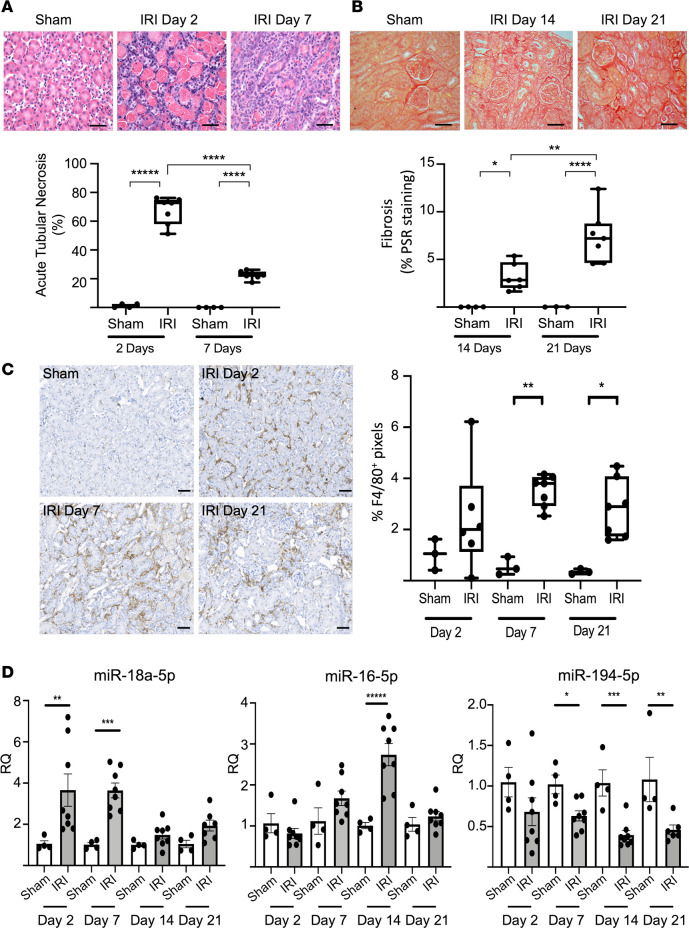
Expression of selected enriched miRNAs in IRI. For the IRI model C57BL/6 mice underwent unilateral renal artery occlusion, resulting in warm ischemia for 18 minutes, followed by reperfusion and recovery for 2–21 days. (**A**) IRI mice demonstrated significant acute tubular necrosis on H&E staining at 2 and 7 days after injury. Scale bar: 50 μm. Data are expressed as median ± min and max value. One-way ANOVA with Tukey’s multiple-comparison test, ****P < 0.0001. (**B**) The normal renal architecture was progressively disrupted with significant fibrosis formation (semiquantified using picrosirius red staining) evident after 2 weeks. Scale bar: 50 μm. Data are expressed as median ± min and max value. ANOVA test with Tukey’s multiple-comparison test, *P < 0.05, **P < 0.01, ****P < 0.0001. (**C**) This injury was accompanied by increased macrophage number (F4/80 staining) as compared with sham animals. Scale bar: 50 μm. Data are expressed as median ± min and max value. ANOVA test with Tukey’s multiple-comparison test, *P < 0.05, **P < 0.01. (**D**) Within the kidney, we noted an upregulation of macrophage-enriched miR-16-5p and miR-18a-5p and downregulation of PT cell–enriched miR-194-5p with injury. Data expressed as mean ± SEM. ANOVA test with Tukey’s multiple-comparison test. *P < 0.05, **P < 0.01, ***P < 0.001, *****P < 0.00001.

To assess if the renal expression of these miRNAs was conserved across renal injury stimuli, we employed a mouse model of ischemia/reperfusion injury (IRI). Following 18 minutes of unilateral renal artery occlusion, the IRI animals demonstrated significant early acute tubular necrosis (ATN), macrophage infiltration, and ultimately progression to fibrosis (Figure 6, A–C). CD68 was used as a macrophage marker, and the staining was positive in the interstitium (Figure 6C), in areas overlapping with those observed for miR-18a-5p and miR-16a-5p (Figure 5B). Macrophage-enriched miR-18a-5p was significantly upregulated as early as 2 days after IRI, and this was followed by miR-16-5p upregulation at 2 weeks (Figure 6D). Conversely, PT cell–enriched miR-194-5p was significantly decreased from 7 days onward (Figure 6D). Although this observation of reduced miR-194 in IRI was consistent with previously published data (7), miR-194 appeared to be a poor early marker of tubular injury because it was unable to detect the significant ATN observed at day 2 (Figure 6, A and D). The dynamic profile of the expression of these miRNAs in IRI bore remarkable similarity to those observed in the R-UUO bulk expression (Figure 5C).

### DEmiRNAs in human renal disease exhibit cell type enrichment.

**Figure 7:**
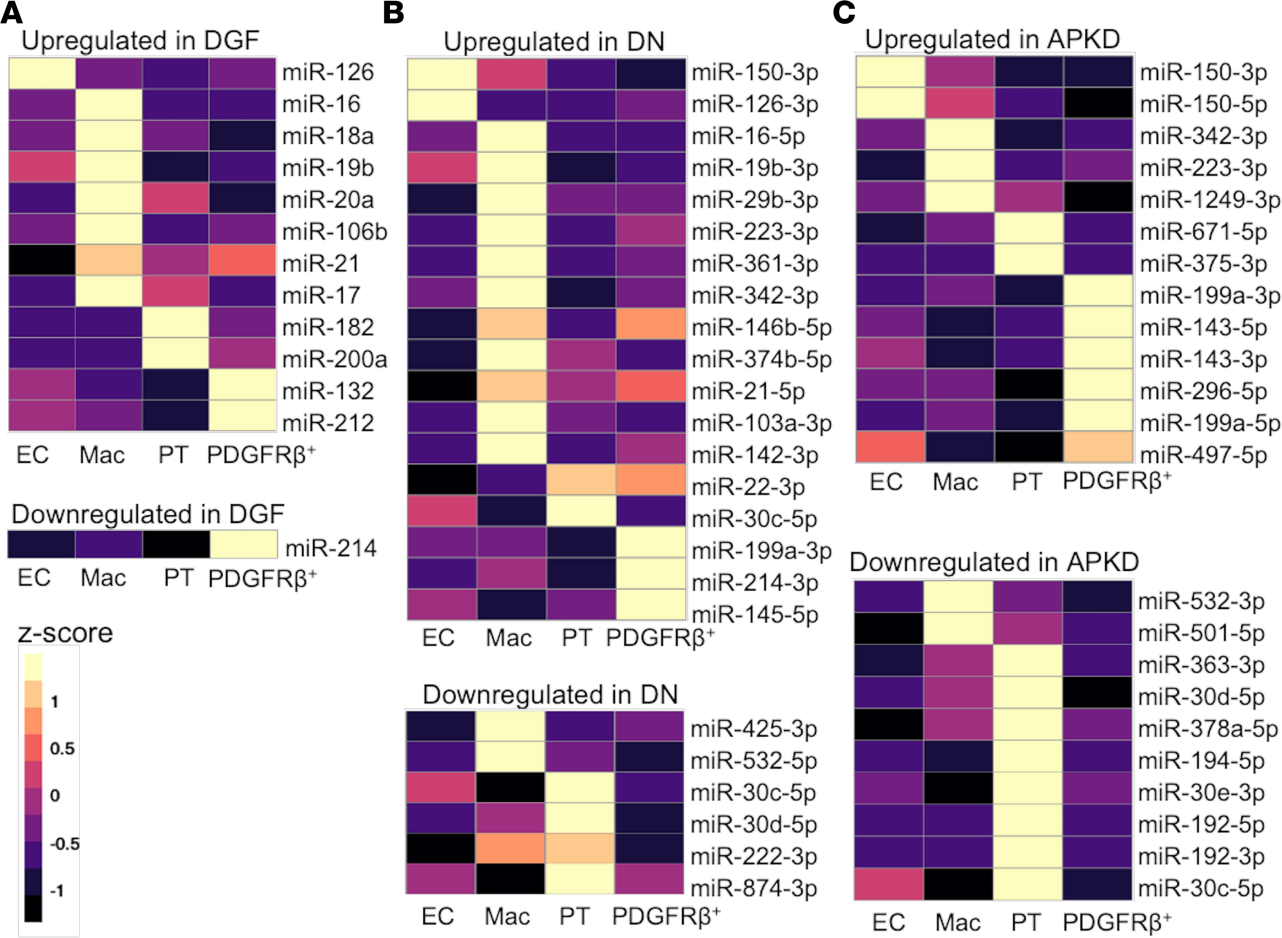
Cell specificity of DEmiRNAs in discrete human renal disease phenotypes. Cell-average expression in each cell type of DEmiRNAs in published human disease data sets: (**A**) delayed graft function (DGF) after renal transplantation, (**B**) diabetic nephropathy (DN), and (**C**) adult polycystic kidney disease (APKD) versus study controls. Mean expression values of miRNAs for each cell type across all conditions were calculated from our sRNA-Seq data. MiRNAs with high enrichment are shown here. Full plots of all DEmiRNAs and details of the data sets used are available in Supplemental Table 2 and Supplemental Figure 12.

We next wished to determine whether cell-specific miRNAs identified in the R-UUO model are implicated in human renal injury. We hypothesized that the application of miRNA enrichment profiles to external bulk sRNA-Seq data sets from patients with kidney disease could increase our understanding of the potential cellular origin of the reported DEmiRNAs. To do this, we profiled the relative expression of each cell-specific miRNA from our data set and crossed this with differentially expressed miRNAs in renal biopsies, from patients with kidney disease (Figure 7, Supplemental Table 2, and Supplemental Figure 12).

We first analyzed the renal miRNA transcriptional changes in human delayed graft function (DGF). DGF is typically the first clinical manifestation of IRI occurring following renal transplantation and histologically is associated with tubular injury and inflammatory cell infiltrates, including macrophages (33, 34). Correspondingly, we identified an upregulation of macrophage-enriched miRNAs (e.g., miR-18a, miR-16) (Figure 7A) when probing the reported DEmiRNAs in DGF.

As diabetic nephropathy (DN) is a leading cause of chronic kidney disease (CKD), we sought to examine the cellular origin of reported DEmiRNAs. We found that miRNAs which were upregulated in kidney biopsies from patients with DN (mean estimated glomerular filtration rate [eGFR] 33 mL/min/1.73 m^2^) compared with controls (eGFR 101 mL/min/1.73 m^2^), corresponded to macrophage-enriched miRNAs (e.g., miR-16, miR-146b) and PDGFR-β^+^–enriched miRNAs (e.g., miR-199a-3p, miR-214-3p), with downregulated miRNAs mapping to PT cell–enriched miRNAs (Figure 7B and ref. 11). This is in keeping with the known inflammatory component of DN, where the intensities of the infiltrates are proportional to the rate of subsequent decline in renal function and tubular loss (15). Similarly, in human polycystic kidney disease — a genetic cause of CKD where healthy tubules are replaced with cysts (10, 35) — the predominant differences are that downregulated miRNAs mapped to the PT-enriched miRNAs (miR-192, miR-194, miR-30 family members), while upregulated miRNAs mapped again to macrophage-enriched miRNAs (e.g., the proinflammatory miR-324) (Figure 7C and refs. 10, 36). In addition, upregulation of PDGFR-β^+^–enriched miRNAs (e.g., miR-199a) was observed, in keeping with the implicated role of myofibroblasts in polycystic kidney disease cyst progression (10, 37).

### Macrophage-enriched miR-18a-5p and -16-5p are early urinary biomarkers of delayed graft function.

Having demonstrated differential expression of miR-18a-5p and miR-16-5p in the kidney of our IRI model and in external data sets of human renal disease, we next wished to assess whether these miRNAs could be detected noninvasively. As a clinical model of acute kidney injury with a significant ischemic etiological component, we studied urine samples from patients with DGF following renal transplantation. DGF is common following deceased donor renal transplantation (38), and the ability to both detect the extent of ischemic injury and predict the likely tempo of recovery would be valuable in tailoring the management of these patients in the early posttransplant period. In order to maximize the potential clinical translation for a point-of-care urinary miRNA test, we employed total urinary RNA quantification after the removal of cellular debris, rather than analysis of individual components, such as urinary exosomes. We hypothesized that changes in macrophage and PT cell–enriched miRNAs from the kidney would be both detectable and differentially expressed when measured in the urine of patients with DGF.

**Figure 8:**
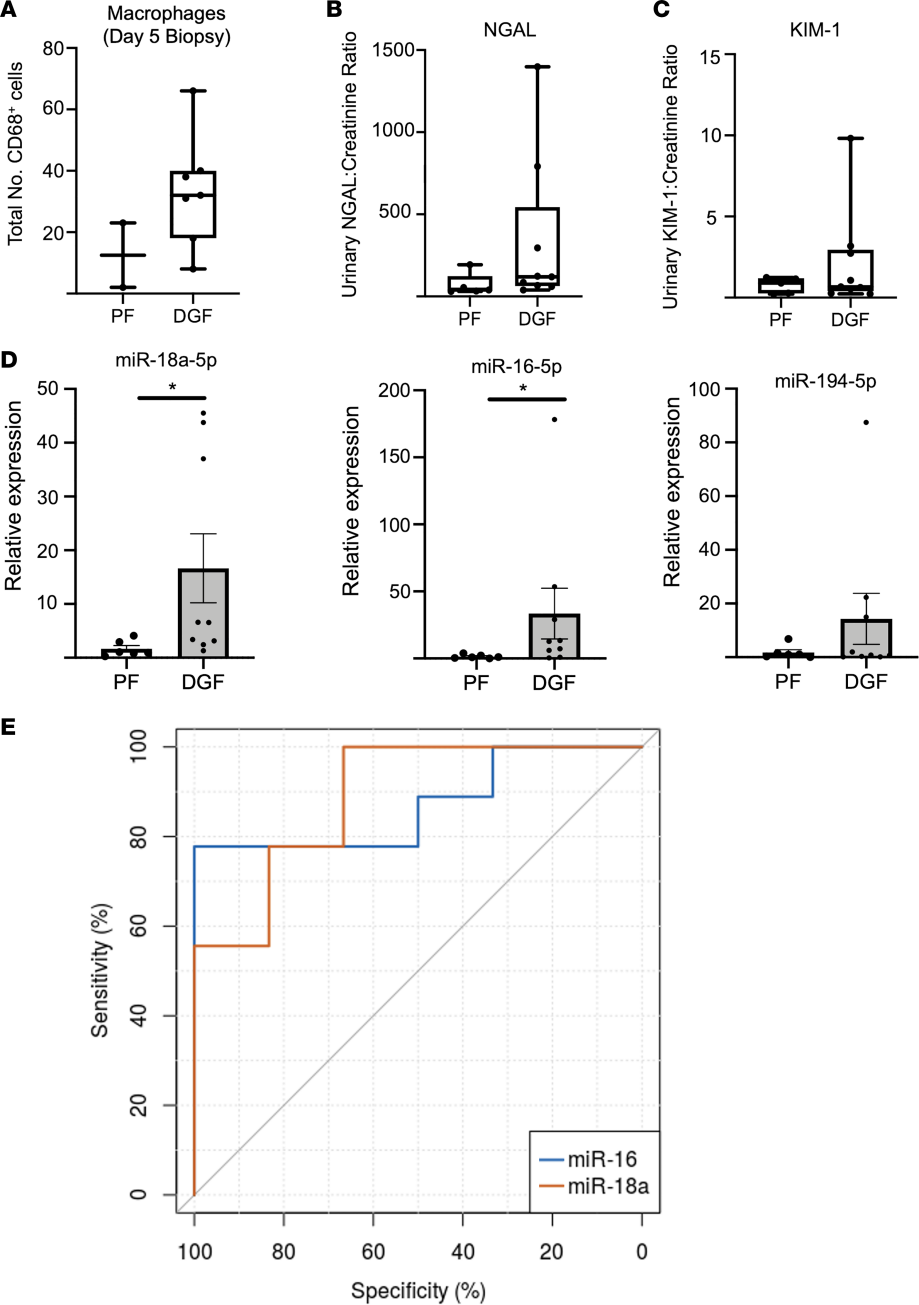
Macrophage-enriched miRNAs 16-5p and 18a-5p are differentially expressed in the urine of renal transplant recipients with DGF. (**A**) On the day 5 renal biopsy, increased macrophage (CD68-positive) cell number was observed in recipients with DGF versus those with PF (*n* = 2). Data are expressed as median ± min and max value. (**B**–**D**) The first available urine sample passed after transplantation was analyzed. No significant difference was seen in urinary injury markers (**B**) neutrophil associated gelatinase lipocalin (NGAL) or (**C**) KIM-1. (**D**) Macrophage-enriched miR-16-5p and miR-18a-5p were increased in recipients developing DGF; miR-194 was unchanged. Data are expressed as median ± min and max value. (**E**) Receiver operating characteristics curve of the differentially expressed miR-16-5p (AUC: 87%) and miR-18a-5p (AUC: 88.9%). Urinary miRNAs were normalized to miR-Cel-39 spike in **D**. Data reported as mean ± SEM. Mann-Whitney *U* test (*P < 0.05). PF, primary function.

Fifteen patients had urine samples available for analysis: 8 (53.3%) of the patients received kidneys from donors after brainstem death (DBD), and 7 donated kidneys (46.7%) were from donors after circulatory death (DCD) (Table 3). There was no significant difference in donor or recipient characteristics, including age, sex, preoperative dialysis, and cause of kidney failure, on univariate analysis. The 30-day and 90-day renal function posttransplant was similar between groups (Table 3). Patients with DGF trended to having increased macrophage (CD68) number on the study protocol biopsy at day 5 (Figure 8A).

Although there was no significant difference in the urinary levels of neutrophil gelatinase-associated lipocalin (NGAL) (Figure 8B) or KIM-1 (Figure 8C), a significant upregulation in miR-16-5p (fold change 11.3, *P* = 0.016) and miR-18a-5p (fold change 6.57, *P* = 0.011) was evident in the first-passed urine of patients who developed DGF versus those with primary function (Figure 8D). No significant change in PT cell–enriched miR-194-5p was noted. Macrophage-enriched miRNAs demonstrated a high discrimination for the prediction of DGF: miR-16-5p (AUC: 87%, CI: 67.9% to 100%) and miR-18a-5p (AUC: 88.9%, CI: 71.3% to 100%) (Figure 8E). MiR-194-5p was a relatively poor predictor of DGF (AUC: 66.7%, CI: 36.6% to 96.72%) (data not shown). The best model performance was achieved by using the combination of miR-16-5p and miR-18a-5p (AUC: 92.6%, CI: 79.3% to 100%). The individual addition of NGAL or KIM-1 to these macrophage-enriched miRNAs did not improve the prediction of the miRNAs.

## Discussion

Understanding the cell specificity of miRNAs’ expression in the kidney is key to unlocking their full potential as therapeutic targets and biomarkers. In this study, we first performed sRNA-Seq on 4 key renal types isolated from the kidney during health, injury, and repair. This foundation allowed us to apply a potentially novel bioinformatic pipeline, establishing miRNA cell-specific enrichment and the consistency of that enrichment throughout renal injury and repair. Applied to murine models, our cell-enriched miRNAs mirrored the renal histology. Furthermore, we were able to deduce the likely cellular origin of DEmiRNAs in bulk sample analyses from external data sets of human kidney disease. Using this data-driven approach, we then rationally identified miRNAs with potential as biomarkers of macrophage expression, finding miR-16 and miR-18a to be consistent markers when translated into another preclinical model of kidney injury, the IRI model. In humans, we then demonstrated that miR-16 and miR-18a are promising urinary markers of early acute kidney injury following renal transplantation. Finally, we made the data freely available through our interactive platform, providing a reference atlas for researchers in the field (http://www.kidney-enriched-micrornas.com).

A number of studies have previously reported kidney-enriched miRNAs, predominantly by microarray profiling of different organs from normal animals. In the original microarray analysis, Sun et al. identified miRNAs -192, -194, -204, -215, and -216 as kidney specific when compared with heart, spleen, muscle, and prostate (16). Other miRNAs, such as miR-30b, -30c, -30d, -10a, and -192 and the miR-200 family, have also been suggested to be kidney specific (39). However, other studies have shown that these miRNAs are expressed in different tissues — for example, miR-192 is expressed in liver hepatocytes (40) — and that they play a role in other conditions, such as cancer (41, 42). Our data suggest this enrichment in the kidney may reflect the cellular makeup of the kidney. We note that the miR-30 family, miR-192, and miR-194 (but not miR-10) are enriched in PT cells, which make up the largest cellular component of the kidney. Correspondingly, in whole-tissue sRNA-Seq, the PT cell–derived miRNAs dominate the expression observed and likely obscure changes in other cell types. MiR-192, -194, and -203 have previously been shown to be enriched in the cortex versus the medulla, consistent with PT specificity. However, the miR-200 family, which were proximal tubule enriched in our analysis, have previously been described as medullary enriched (43). Thus, a caveat of our approach is that it may not be capable of discriminating between different portions of the nephron, as some miRNAs may be expressed throughout the EC compartment at differing relative abundance.

A key strength in our approach over other studies is that it does not assume that miRNAs that are enriched in healthy animals will remain specific despite the transcriptomic changes evoked during injury and repair. In fact, in our study only 31.6% of enriched miRNAs maintained cell-specific enrichment. This affects our mechanistic understanding of the cellular origin of miRNA when comparing control versus injury using bulk transcriptomics. Our stringent approach has resulted in overlap with other studies, offering support for their enrichment findings. For example, data from Lorenzen et al. described miR-24 as being endothelial enriched (44), a finding supported by our study. Furthermore, we confirm that the PDGFR-β^+^–enriched miRNAs described by Nakagawa and colleagues — including miR-214-3p, -199a-5p, and -199a-3p — are highly PDGFR-β^+^–enriched in our data set (45). Interestingly, the miR-21 family appeared highly enriched in macrophages in our analyses. Thus, the relatively consistent observation of raised miR-21 with renal injury (46–48) could in part be related to inflammatory cell infiltrates when examining bulk renal samples. The ability to scrutinize the relative miRNA abundance in each cell type using this data set is an advantage over lengthy in situ staining procedures to assess the cellular origin of miRNAs.

Our discovery of miR-16-5p and 18a-5p as potential urinary biomarkers of DGF is a potentially unique finding. These miRNAs have been described before in the kidney and other cell types. MiR-18a-5p has been shown to be upregulated in rat IRI kidney and downregulated in the whole blood (49). In addition to being biomarkers of disease, they may be therapeutic targets. Overexpression of miR-16 in models of atherosclerosis has been reported to reduce proinflammatory macrophage cytokine release, such as IL-6, while promoting the expression of antiinflammatory IL-10 (50). Conversely in the liver, miR-18a induces an M1 proinflammatory macrophage status (51). Our finding of a reduction in miR-194 in the kidney following IRI is in keeping with findings from Godwin et al. (7). They believed their signature to be independent of infiltrating cells by showing this to be maintained in R^o^/cγ0^–^ deficient mice that lack NK cells, B cells, and T cells. In future, larger prospective clinical studies with multivariate regression modeling would be required to determine the accuracy and generalizability of these miRNA markers when applied to DGF or non–transplant-associated acute kidney injury, where there is a clinical need for improved markers.

Our approach, although comprehensive for the technology available, is limited to 4 cell types as is dictated by the FACS technology. While we chose to focus on cell types known to play a critical role in renal injury, we acknowledge other cell types do play a role. At present single-cell miRNA technology remains limited in its scope compared with scRNA-sequencing for mRNA. It is often limited to characterizing a small number of miRNAs, which may introduce more bias (52, 53). More recently, a half-cell single-cell sequencing approach has been utilized by Wang et al. (54). However, this method was very low-throughput, precluding its use in in vivo modeling of kidney disease. In future, should scRNA-sequencing technology become practical for in vivo profiling of the miRNAs in the kidney, our study will provide an invaluable reference to aid in miRNA cluster classification.

In summary, in this study we define the cellular specificity of miRNAs in the normal, injured, and repairing kidney. We demonstrate that consistent cell enrichment during injury and repair is observed only for selected miRNAs. We were able to apply this knowledge to unravel the cellular origin of DEmiRNAs in bulk kidney samples and rationally select macrophage-enriched miRNAs as urinary acute kidney injury biomarkers. Future studies could expand our findings and add various other cell types, which could be integrated to generate a full sRNA-Seq atlas of the kidney in injury and repair.

## Methods

### Murine models

All protocols and surgical procedures were performed following approval of and in accordance with the local animal care committee and Animal Scientific Procedures Act 1986.

#### R-UUO model.

The R-UUO procedure was performed as previously described (20). Male 8- to 12-week-old C57BL/6 mice (Envigo) (*n* = 5/group) underwent laparotomy and distal ureteric dissection. To induce UUO, the ureter was ligated twice with 6/O silk suture distally, close to the bladder. A silastic tube was placed around the proximal ureter to prevent excessive dilatation, which would impede ureteric reimplantation for the R-UUO group. In the R-UUO group, the ureter proximal to the ligature was reimplanted into the bladder following 7 days of obstruction (UUO). Mice were culled at day 2 after UUO (UUO-2), at day 7 after UUO (UUO-7), and in the R-UUO group at 7, 14, or 21 days after reversal by exsanguination under terminal isoflurane anesthesia. Based on data from previous experiments, we determined that *n* = 5/group was sufficient to provide 80% power to detect 20% difference in the degree of renal fibrosis between groups, but we performed sequencing on *n* = 4 animals that had the closest to mean KIM-1/Havcr1 expression.

#### IRI model.

Male C57BL/6 mice (8–10 weeks old, Envigo) underwent laparotomy, renal vascular pedicle exposure, and unilateral renal artery occlusion (ischemia) for 18 minutes followed by reperfusion. Culls were performed at 2, 7, and 14 days following IRI (*n* = 8 per group). Sham animals similarly underwent renal pedicle exposure without arterial clamping and were culled at matched time points (*n* = 4 per group). Based on pilot data from previous experiments, we determined this was sufficient to provide 90% power to detect 25% difference in the degree of renal fibrosis between sham and IRI groups. At the termination of an experiment, mice were culled under isoflurane terminal anesthesia and confirmed using cervical dislocation.

### Kidney tissue processing

After culling, a thoracolaparotomy was performed, the right atrium was punctured, and 5 mL saline was infused via a left ventricular puncture in order to flush the kidneys prior to renal harvesting. Kidneys were divided into segments and either processed immediately (e.g., for FACS experiments), snap frozen, and stored at –80°C or fixed in either 10% formalin (MilliporeSigma) to produce FFPE blocks or IHC zinc fixative for ZnFPE (BD Biosciences). Fixed samples were subsequently placed into 70% ethanol prior to being embedded in paraffin blocks.

### Histology

FFPE or ZnFPE kidney blocks were cut into 3 μm sections. H&E staining was performed on FFPE sections. Sections were rehydrated and stained with hematoxylin (MilliporeSigma) for 5 minutes and washed in running tap water. Slides were dipped in 1% acid alcohol, washed in tap water then Scott’s tap water, and then counterstained in eosin solution (MilliporeSigma). Picrosirius red staining was performed on deparaffinized and rehydrated FFPE sections. Slides were stained with Picro-Sirius Red Solution (ab150681, Abcam) in the dark for 60 minutes. Slides were rinsed twice with acetic acid solution, dehydrated with 100% ethanol, and mounted. For the F4/80 staining, 3 μm ZnFPE blocks were deparaffinized and rehydrated. Antigen unmasking was performed using citric acid (10 mM citric acid, 0.05% Tween-20, pH 6.0). Slides were blocked for endogenous peroxide with 0.2% H_2_O_2_, loaded in Sequenza racks (Thermo Fisher Scientific), and rinsed with PBS. Endogenous avidin and biotin were blocked with Avidin/Biotin Blocking Kit (Thermo Fisher Scientific). Slides were further blocked in Trident Universal Protein Block (GeneTex, Insight Biotechnology). Primary antibody (F4/80, clone BM8, ab16911, Abcam) was incubated at 1:100 overnight at 4°C. After incubation slides were washed with PBS and biotinylated goat anti-rat (Vector Laboratories, 2BScientific Ltd) at 1:200 diluted in antibody diluent (Vector Laboratories). Slides were washed and incubated with ABC Elite (Vector Laboratories), then DAB + (Agilent Technologies). Slides were then counterstained with Harris Haematoxylin (MilliporeSigma), then dehydrated through ethanol gradient and mounted with Pertex (MilliporeSigma).

### In situ hybridization for miRNA localization

FFPE kidney blocks were cut into 3 μm sections. Sections were baked at 60°C overnight and then deparaffinized and rehydrated. In situ hybridization was carried out using miRCURY LNA miRNA probes (Qiagen) for miR-194-5p, miR-16-5p, and miR-18-5p and a control scramble probe. All probes were 5′, 3′ FAM labeled, and in situ was carried out as described in the miRCURY LNA miRNA ISH Optimization Kits (FFPE) handbook with the following changes. Following rehydration sections were antigen unmasked using 10 mM sodium citrate buffer, washed, and incubated with 0.1% Triton X-100 PBS for 10 minutes and washed prior to proteinase K treatment. The probes were hybridized overnight at hybridization Tm _RNA_ and the NBT-BCIP solution left on for 5 hours. Images were taken on an ECLIPSE Ci-L bright-field microscope (Nikon) and the DS-L4 imaging program.

#### Kidney digestion for FACS.

Immediately after culling, mice were perfused with 5 mL PBS. The kidneys were harvested, with the renal capsule removed and placed in ice-cold PBS. Equivalent kidney sections were finely minced in digest buffer (4.25 mg/mL Collagenase V from MilliporeSigma, 6.25 mg/mL Collagenase D from Roche, 10 mg/mL Dispase from Thermo Fisher Scientific, and 300 μg/mL DNase from Roche in RPMI 1640 with 10% FCS from Thermo Fisher Scientific, with 1% penicillin/streptomycin/l-glutamine from Thermo Fisher Scientific) prior to homogenization in gentle MACS C-tubes using the gentle MACS dissociator (Miltenyi Biotec). Samples were incubated at 37°C with shaking to maximize digestion. The kidney suspension was subjected to a second gentle MACS homogenization and digestion neutralized with an equal volume of FACS buffer (PBS, 2 mM EDTA, and 2% FCS). Kidney cell suspensions were then sequentially passed through 100 μm, 70 μm, and 40 μm sieves. Any residual RBCs were lysed by RBC lysis buffer (MilliporeSigma). Cells were resuspended in ice-cold FACS buffer ready for use.

#### Fluorescence-activated cell sorting.

Cell suspensions were incubated with FC block (BD Biosciences) and subsequently incubated with preconjugated antibodies in a round-bottomed plate. Controls were set up including unstained cells, beads with single stains of each antibody, and fluorophore minus one. DAPI (MilliporeSigma) (1:1000) was added to samples immediately prior to acquisition to assess cell viability. Cells were sorted for 4 positive cell type populations (PT [LTL], Mac [F4/80], endothelial [CD31], and fibroblast [PDGFR-β] using antibodies detailed in Table 4.

### RNA extraction methods

#### RNA extraction from FACS populations.

Following FACS, cells were sorted into RLT buffer (Qiagen) at room temperature. RNA was extracted using RNeasy Micro Kit (Qiagen), which has been amended for miRNA extraction. Briefly, cells were homogenized by vortexing for 10 seconds; the lysate was pipetted onto a gDNA Eliminator spin column and centrifuged at 10,000*g* for 30 seconds. The gDNA column was discarded and 1.5 times volume molecular grade pure ethanol was added to the lysate and placed on an RNeasy MinElute column. The column was spun at 10,000*g* for a further 30 seconds, 700 mL RWT buffer was added, and the extraction continued as per manufacturer’s protocol.

#### RNA extraction from tissue.

Total RNA from cortical kidney tissue was obtained using the miRNeasy kit (Qiagen) following the manufacturer’s instructions. Tissue was lysed using 700 mL QIAzol and homogenized with steel beads (Qiagen) using Tissue Lyser II (Qiagen) for 1 minute.

#### RNA extraction from urine.

RNA was extracted using the miRNeasy Mini Kits (Qiagen) with the following modification to the manufacturer’s instructions; 750 mL of QIAzol lysis reagent was added along with 1 mg carrier RNA (MS2 RNA, Roche). For normalization of the sample, synthetic *Caenorhabditis elegans* cel-miR-39 (MC10956, cel-miR-39; Qiagen) was spiked in to achieve a final concentration of 0.5 pM. Thereafter the miRNeasy manufacturer’s instructions were followed (46).

#### RNA-sequencing.

RNA integrity was checked using Agilent Technologies nanochips. All samples utilized for sRNA-Seq had a minimum RNA integrity score of 7. For the single-population sRNA-Seq, RNA underwent sRNA-Seq by Genewiz, using the Illumina HiSeq platform, generating paired-end reads of 150 bp (*n* = 4 per group). For the whole-tissue sRNA-Seq, RNA underwent sRNA-Seq by Genewiz, using the Illumina HiSeq 2500 platform, generating single-end reads of 50 bp performed over 2 runs (*n* = 4 per group). The sequencing data are deposited with the National Center for Biotechnology Information’s Gene Expression Omnibus database, accession number GSE150035.

### Bioinformatics analysis

#### Alignment and preprocessing.

Raw reads were examined for quality control by FastQC. Raw reads were trimmed using TrimGalore (Ver 0.4.1)/ Cutadapt (version 1.1) to remove primer/adaptor contamination and low-quality reads (<20 Phred score). Clean, high-quality, trimmed reads as confirmed by FastQC were aligned to the mouse genome GRCm38 and MiRBase (Ver 22) using the Bowtie wrapper Shortstack (55), achieving a mapping rate of 72% to 86% in the single-population sRNA-Seq. Alignment rates between cell types and experimental conditions were similar. This provided a count table of miRNA reads ready for downstream analysis in Rstudio (Ver 3.6.1). The pipeline for the whole-tissue sRNA-Seq was as above, except the 2 runs of the sequencing were merged before entering the pipeline. For preprocessing, the R package edgeR was employed to generate counts per million, lowly expressed reads were removed, and normalization for library size was performed (56).

#### Differential expression.

Exploratory principal component analyses were performed using DESeq2 (Version 1.24.0) (57). This revealed sample 68c was a significant outlier (PDGFR-β group, UUO-7), and as such this sample was removed from all downstream analyses (Supplemental Figure 1B), leaving 3 samples for this group. Otherwise for each cell type and condition, there were 4 samples per group. Differential expression analysis was performed on normalized counts principally using EdgeR (Version 3.26.8) (56). EdgeR uses a negative binomial distribution to model miRNA reads in deep sequencing data sets. A fold change > 1.5 and FDR (Benjamini-Hochberg method) < 0.05 were considered significant. Data are freely available at http://www.kidney-enriched-micrornas.com Clusters of dynamic expression within each cell type were curated by generating logical arguments based on the differential expression analysis (Supplemental Table 3). Within this system, a miRNA could not exist in multiple clusters. Validated gene targets of enriched miRNAs were identified using the R package multimiR (Version 1.6.0), which searches “mirecords,” “mirtarbase,” and “tarbase” (58).

#### Cell enrichment clustering.

Unbiased fuzzy clustering was performed for each experimental condition using the R package mFuzz (Version 2.44) (24) on normalized counts. This approach has the advantage of being more robust to noise and allows the global clustering structure to be visualized. A membership value of 1.18 and 16 clusters was utilized based on the protocol guidelines. Clusters that demonstrated an increased expression in a single cell type for more than 2 samples were selected as potential enriched clusters and taken forward. This process was repeated until mFuzz cell type clusters for sham, UUO-2, UUO-7, and R-UUO were obtained.

A limitation of fuzzy clustering in the context of identifying a cell-specific miRNA is that a miRNA could exist in multiple clusters. Therefore, in order to improve cell specificity, this unbiased fuzzy clustering was combined with selecting miRNAs based on their relative cell type expression. For each condition, the mean cell type–specific *z* score of an individual miRNA was calculated, and miRNAs with a *z* score more than 1.15 were considered cell type enriched by this approach. The *z* score represents the number of SDs the expression of a miRNA in a sample is away from the mean of all the values for the same miRNA. The *z* score is equal to: (log[sample miRNA expression] – mean[log(miRNA expression)])/SD. This cutoff was selected because, at this level of stringency, stable cell type clusters were evident when the data were reclustered using agglomerative hierarchical clustering by Euclidian distances (using pHeatmap, Version 1.0.12).

MiRNAs enriched using both approaches were taken forward as cell-enriched markers for each time point. MiRNAs that demonstrated enrichment for more than one cell type were considered Not Cell Specific. Enrichment consistency was considered low, moderate, or high based on the number of experimental conditions (out of 4) the miRNA demonstrated cell type enrichment (1, 2–3, 4, respectively).

#### Enrichment comparisons in external data sets.

We wished to explore the likely cellular origin of DEmiRNAs published in murine and human renal diseases identified from predominantly high-throughput approaches. We gave preference to studies which (a) were in humans, (b) were published recently, and (c) had data available within the supplementary materials or deposited on GEO2R.

Many of the studies encompassed microarray data and older studies where the nomenclature of miRNAs had changed or where the miRNA strand information was not available. In addition, there are considerable inconsistencies in between the nomenclature of murine and human miRNA annotation. To address this, we first employed the R package miRBaseConverter (Version 1.8.0) (59) to determine the annotation of the external data set and convert it to the latest miRBase version (version 22) as used in our study. For the human to mouse miRNA annotation matching, we obtained the species-specific mature miRNA sequences from miRbase (60). We then matched the murine to human sequences on the basis of the same seed sequence (nucleotides 2 to 7) and similarity of miRNA name. This generated 476 miRNAs with a unique seed sequence and miRNA name paired for mouse and human that were used for annotation merging of the data sets.

### Human subjects

Analysis was performed on stored samples from renal transplant recipients previously recruited to a clinical trial (ClinicalTrials.gov NCT01430156) investigating delayed graft function (61). The full details of the study are as reported by Thomas et al. (61). We included patients recruited to the placebo arm of this trial who had urine samples stored and available for analysis. DGF was defined as “increased or stable serum creatinine, or a decrease of less than 10% per day for 3 consecutive days in the first week after transplantation” (61). We took advantage of measurements of urinary KIM-1 (ng/mL) and NGAL (ng/mL), which had been quantified by ELISA, and normalized these measurements to urinary creatinine to correct for differences in urinary dilution. Renal biopsies were obtained and studied as part of the trial on day 5 following transplant. Renal macrophages were identified by immunofluorescence using anti-CD68 antibody (ab13243, Abcam). The number of macrophages was quantified by counting CD68-positive cells in 8 nonoverlapping fields at ×20 magnification on a ZEISS LSM 710 confocal microscope.

### Urinary samples and miRNA measurements

Urine samples were obtained on day 0 (day of transplant) and days 1, 2, 3, and 5 posttransplant if available. Urine samples were stored at –80°C for further analysis. Following collection, urine samples were stored at –80°C. Samples were defrosted and centrifuged at 2000*g* for 10 minutes at 4°C to remove any cellular debris. The top 350 mL was taken as the sample and RNA extracted as described above.

### MiRNA and gene expression analysis

FACS-sorted cells were collected for 4 positive cell populations. cDNA was synthesized from 750 pg of template RNA using the Ovation RNA Seq V2 (Nugen) amplification as per manufacturer’s instructions. Gene expression was assessed by qRT-PCR using the PerfeCTa FastMix II probe master (VWR) and TaqMan Gene Expression assays (Life Technologies, Thermo Fisher Scientific) and normalized to hypoxanthine-guanine phosphoribosyltransferase (HPRT Mm00446968_m1).

For quantitative real-time PCR analysis of gene expression, cDNA was synthesized from 1 µg of template RNA, using the QuantiTect Reverse Transcription Kit (Qiagen). Quantitative PCR was performed using the PerfeCTa FastMix II probe master (VWR) and TaqMan Gene Expression Assay–specific primers (Life Technologies, Thermo Fisher Scientific) for Havcr1 (Mm00506686_m1) and normalized to hypoxanthine-guanine phosphoribosyltransferase (HPRT Mm00446968_m1).

For quantitative real-time PCR analysis of miRNA expression, reverse transcription of total RNA was performed with Applied Biosystems TaqMan MicroRNA Reverse Transcription Kit (Life Technologies, Thermo Fisher Scientific), followed by quantitative PCR using Universal Mastermix II (no UNG) (Life Technologies, Thermo Fisher Scientific) and specific miRNA assays (miR-18a-5p 002422, miR-16-5p 000391, miR-194-5p 000493, U6 001973).

Normalization was to endogenous U6 and exogenous spike miR-cel-39 for the analysis of murine kidney and human urine samples, respectively. The relative quantification of miRNA differential expression was calculated using the 2−ΔΔCT method.

### Statistics

Statistical analyses were performed using either GraphPad Prism 8.0 or specific packages within R (3.6.1) for more complex analyses. Two independent samples were compared using the Mann-Whitney *U* test.

For 3 or more independent samples, 1-way ANOVA was used, and when equality of variances could not be ensured, Welch’s 1-way ANOVA was substituted. Adjustments for multiple testing were included using the Tukey or Dunnett methods as appropriate, and all tests were 2 tailed.

Receiver operating characteristics were calculated using the R package pROC (Version 1.16.2) (62). To assess the prediction of urinary miRNAs in combination, a multivariable binomial logistic regression model was performed in R using the glm wrapper finalfit (Version 1.0.1). Kolmogorov-Smirnov tests were used to compare the probability distribution curves of the global log fold changes of enriched miRNAs. All other statistical analyses performed as part of the sRNA-Seq analysis are outlined in the corresponding sections.

A statistical probability *P* < 0.05 was considered significant. Significance is depicted as follows: *P < 0.05, **P < 0.01, ***P < 0.001, and ****P < 0.0001.

### Study approval

#### Murine models.

All protocols and surgical procedures were performed following approval and in accordance with the local animal care committee at the University of Edinburgh and the Animal Scientific Procedures Act 1986.

#### Human participants.

With approval from the trial team and study sponsor Academic and Clinical Central Office for Research and Development, analysis was performed on biobank samples collected as part of the clinical trial (ClinicalTrials.gov NCT01430156) (61) in accordance with the Declaration of Helsinki. Written informed consent was received from participants prior to inclusion in the trial, including the use of samples for further research. The full details of the study are as reported by Thomas et al. (61).

## Author contributions

KLC conceived the study, designed the methodology, investigated, analyzed data, and wrote and edited the manuscript. LD acquired funding, supervised the study, conceived the study, performed project administration, designed methodology, investigated, analyzed data, and wrote and edited the manuscript. SJW, LPM, BRC, and JH acquired funding, supervised the study, conceived the study, and edited the manuscript. CC investigated, acquired data, analyzed data, and wrote the manuscript. OT, VB, RABT, and SF investigated, designed experimental methodology, acquired data, and analyzed data. JR, RP, and EMH analyzed data and designed bioinformatic methodology. GMT, RBH, COSS, and VS acquired funding, conceived the study, and performed project administration.

## Supplementary Material

Supplemental data

Supplemental Data Set 1

## Figures and Tables

**Table 3:**
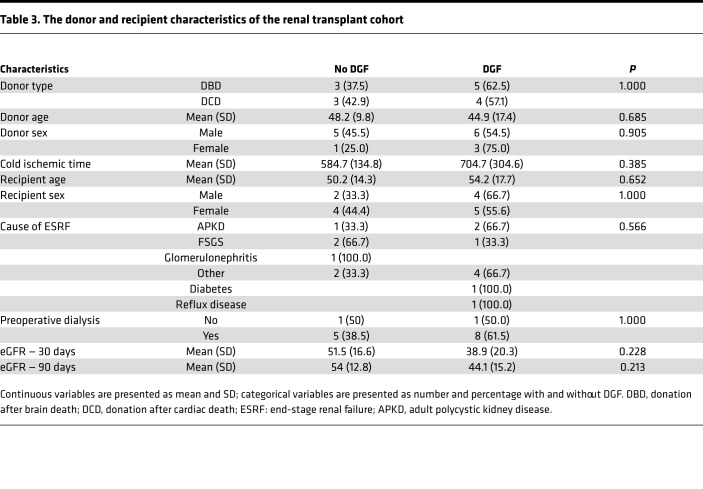
The donor and recipient characteristics of the renal transplant cohort

**Table 1:**
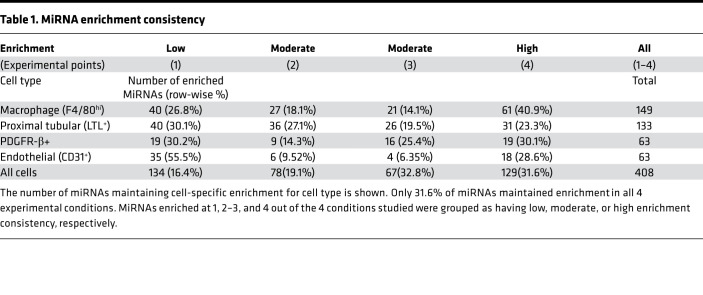
MiRNA enrichment consistency

**Table 2:**
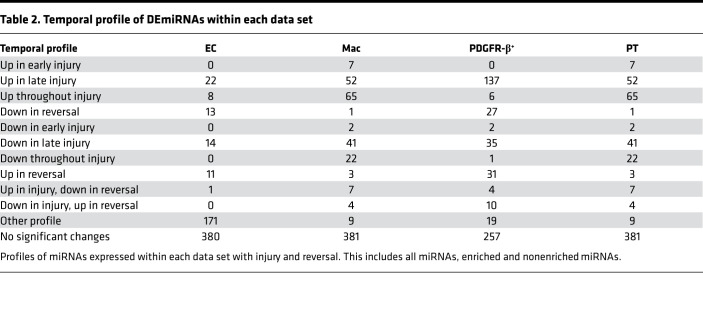
Temporal profile of DEmiRNAs within each data set

**Table 4:**
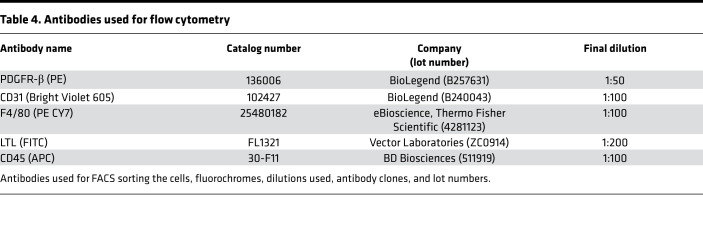
Antibodies used for flow cytometry

## Version 1 12/17/2020

Electronic publication
