# Supplementary material for: Identifying cell-enriched miRNAs in kidney injury and repair
Source: JCI Insight. 2020 Dec 17;5(24):e140399. doi: 10.1172/jci.insight.140399 (PMC7819746; doi:10.1172/jci.insight.140399)
Supplement: Supplemental data [file jciinsight-5-140399-s057.pdf]

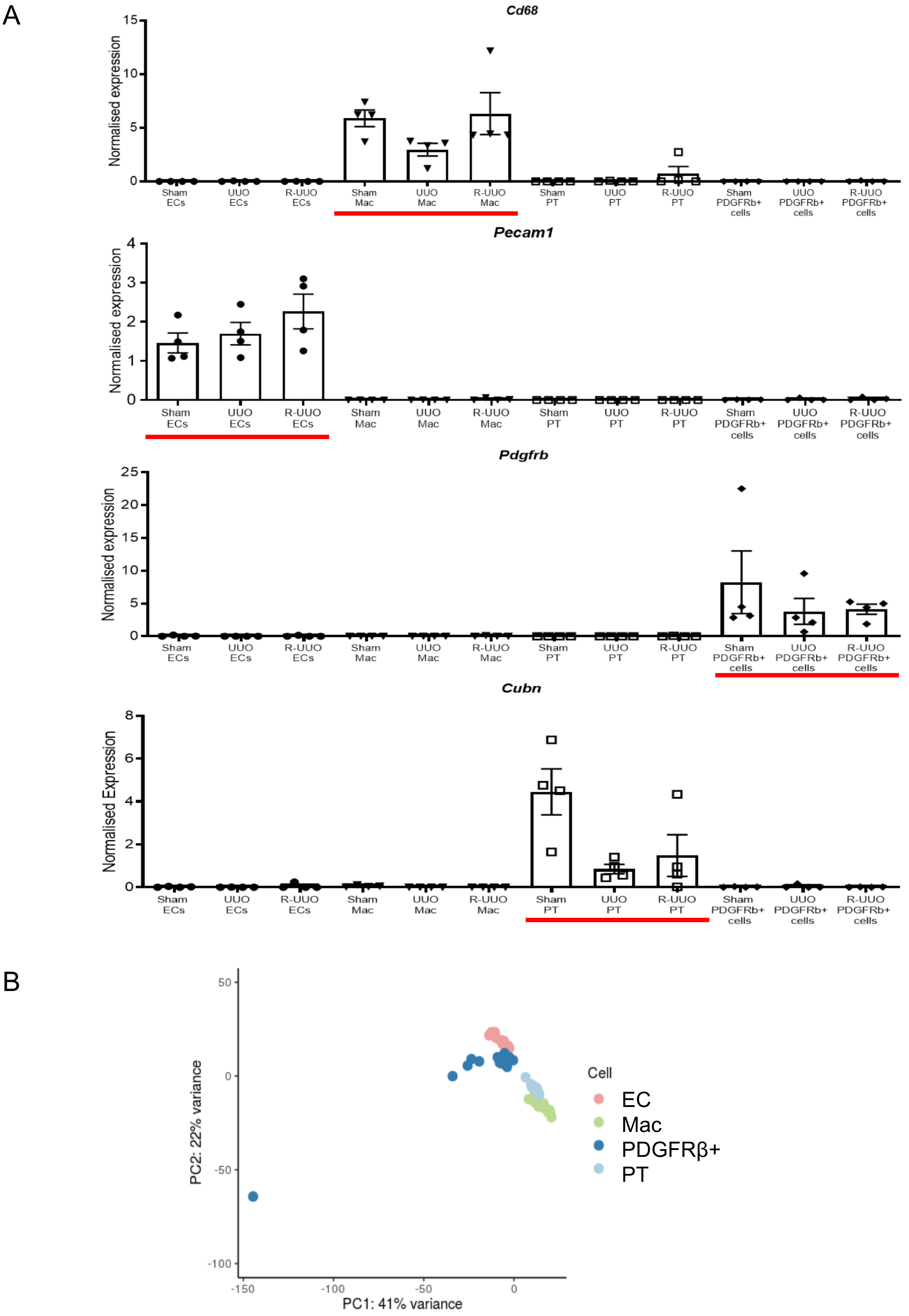

Supplementary Figure 1: Quality control of FACS sorted cell populations and small-RNA sequencing. A: qRT-PCR for DEmarker genes for each single population type. Red line highlights cell type for DEmarker gene (n=4/gp). **B:** Principal component analysis of all sRNA samples revealed a sample from the PDGFRβ⁺ UUO-7 group to be a significant outlier. This was sample was excluded from downstream analyses. Macrophage (Mac), Endothelial Cells (EC), and Proximal Tubules (PT).

A

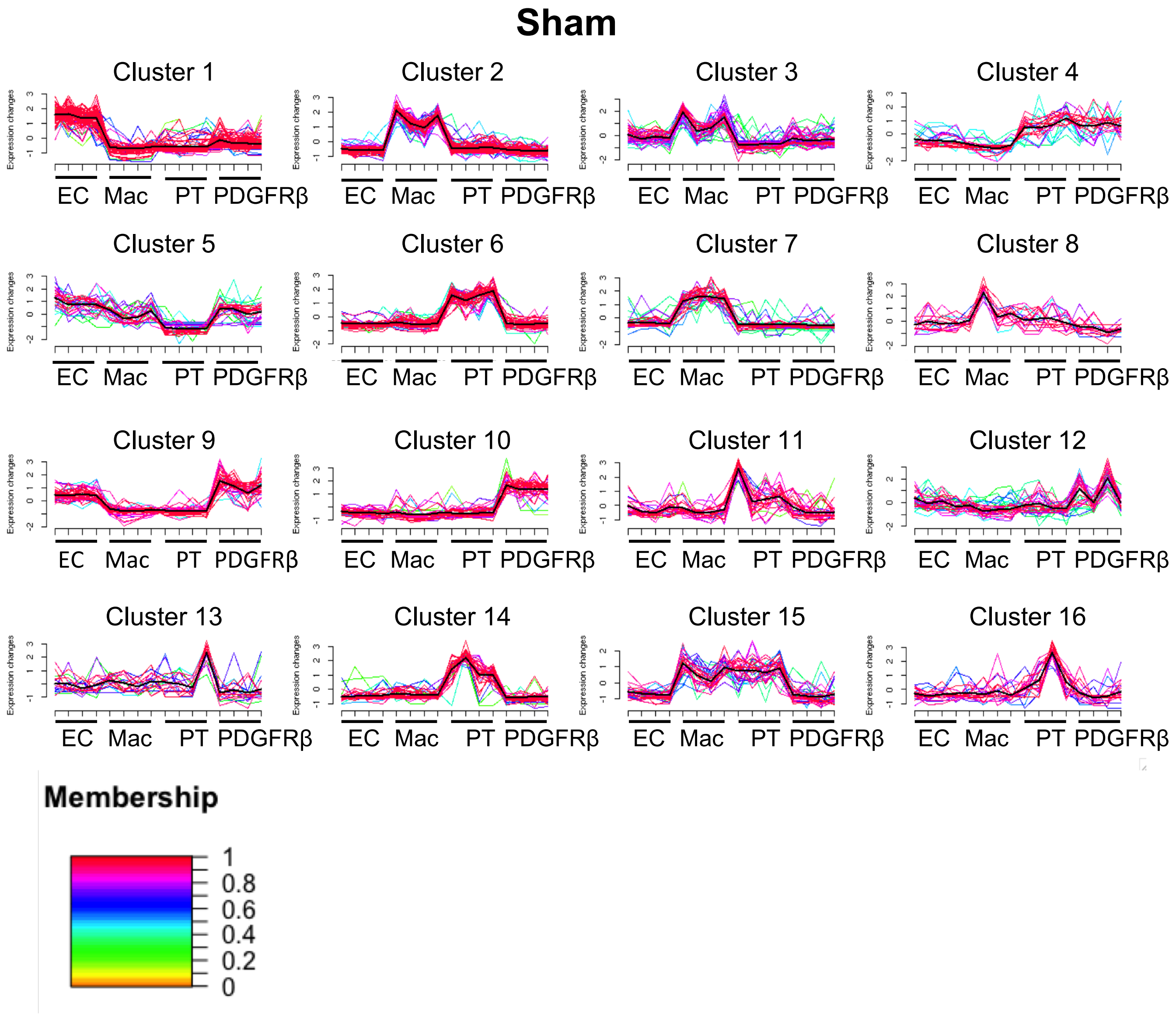

B

Cell Enrichment	Sham mFuzz Cluster(s)
Macrophage (F4/80^{hi})	2, 3, 7
Proximal Tubular (LTL^{+})	6, 14
Fibroblast (PDGFRβ^{+})	10
Endothelial (CD31^{+} CD45^{-})	1
Enriched in multiple cell types	4, 5, 8, 9, 11, 12, 13, 15, 16

Supplementary Figure 2: Mfuzz clustering of the single population data from Sham timepoint.
A: Clustering of single population sRNA-seq microRNAs by mFuzz demonstrates cell specific clusters (expression changes >0 in one cell type for >2 samples) for each of the isolated populations. The membership value colour indicates how well an individual expression pattern in an MFuzz cluster fits the dominant pattern in that cluster. Each downward tick on the x-axis represents a sample (n=4 for each cell type). **B:** Assignment of an mFuzz cluster to an enriched profile. mFuzz Parameters: Clusters 16, membership score 1.18.

A

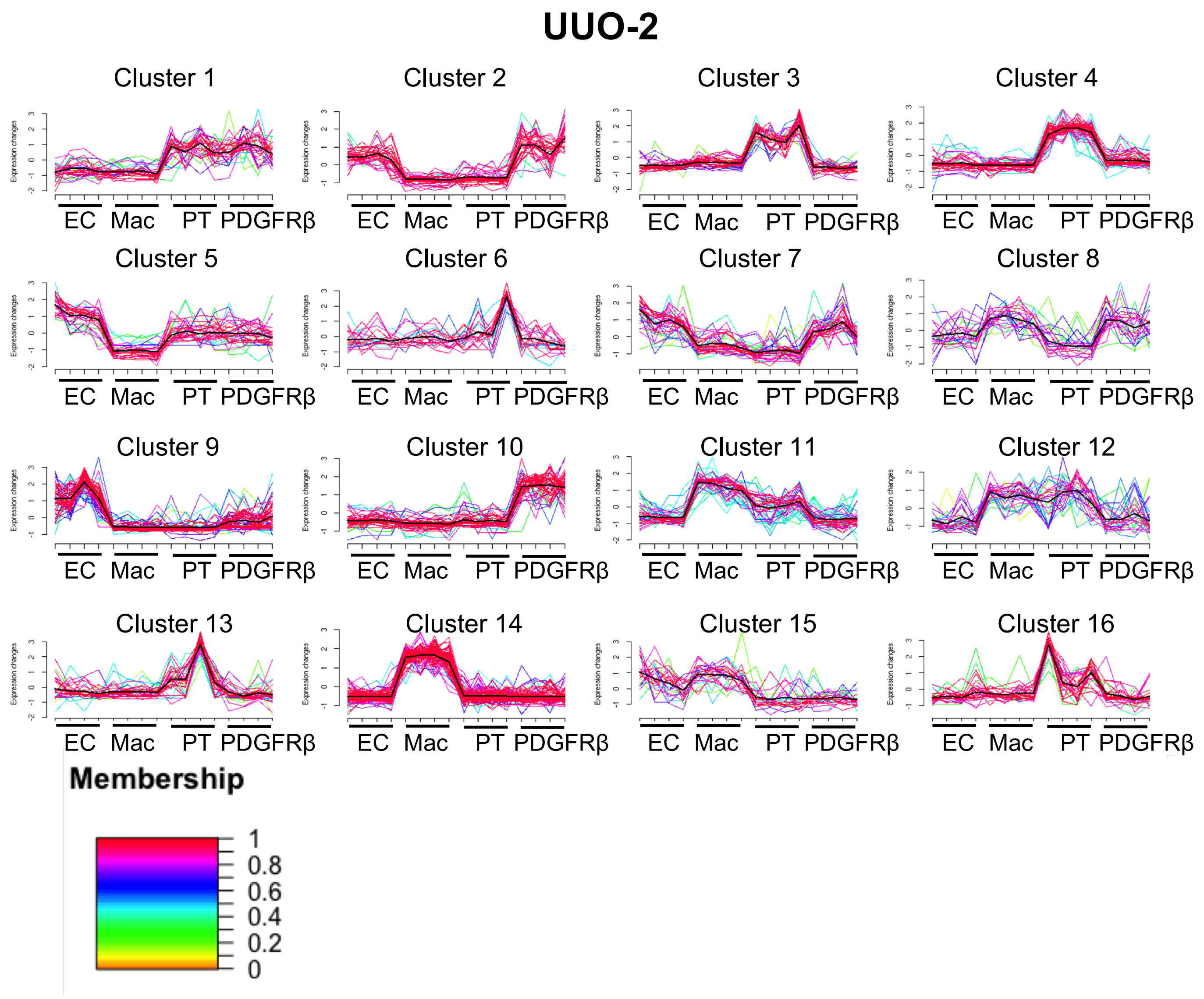

B

Cell Enrichment	UUO-2 mFuzz Cluster(s)
Macrophage (F4/80^{hi})	14
Proximal Tubular (LTL^{+})	3, 4, 13
Fibroblast (PDGFRβ^{+})	10
Endothelial (CD31^{+} CD45^{-})	9
Not enriched by a single population	1, 2, 5, 6, 7, 8, 11, 12, 15, 16

Supplementary Figure 3: Mfuzz clustering of the single population data from UUO-2 timepoint.
A: Clustering of single population sRNA-seq microRNAs by mFuzz demonstrates cell specific clusters (expression changes >0 in one cell type for >2 samples) for each of the isolated populations. The membership value colour indicates how well an individual expression pattern in an MFuzz cluster fits the dominant pattern in that cluster. Each downward tick on the x-axis represents a sample (n=4 for each cell type). **B:** Assignment of an mFuzz cluster to an enriched profile. mFuzz Parameters: Clusters 16, membership score 1.18.

A

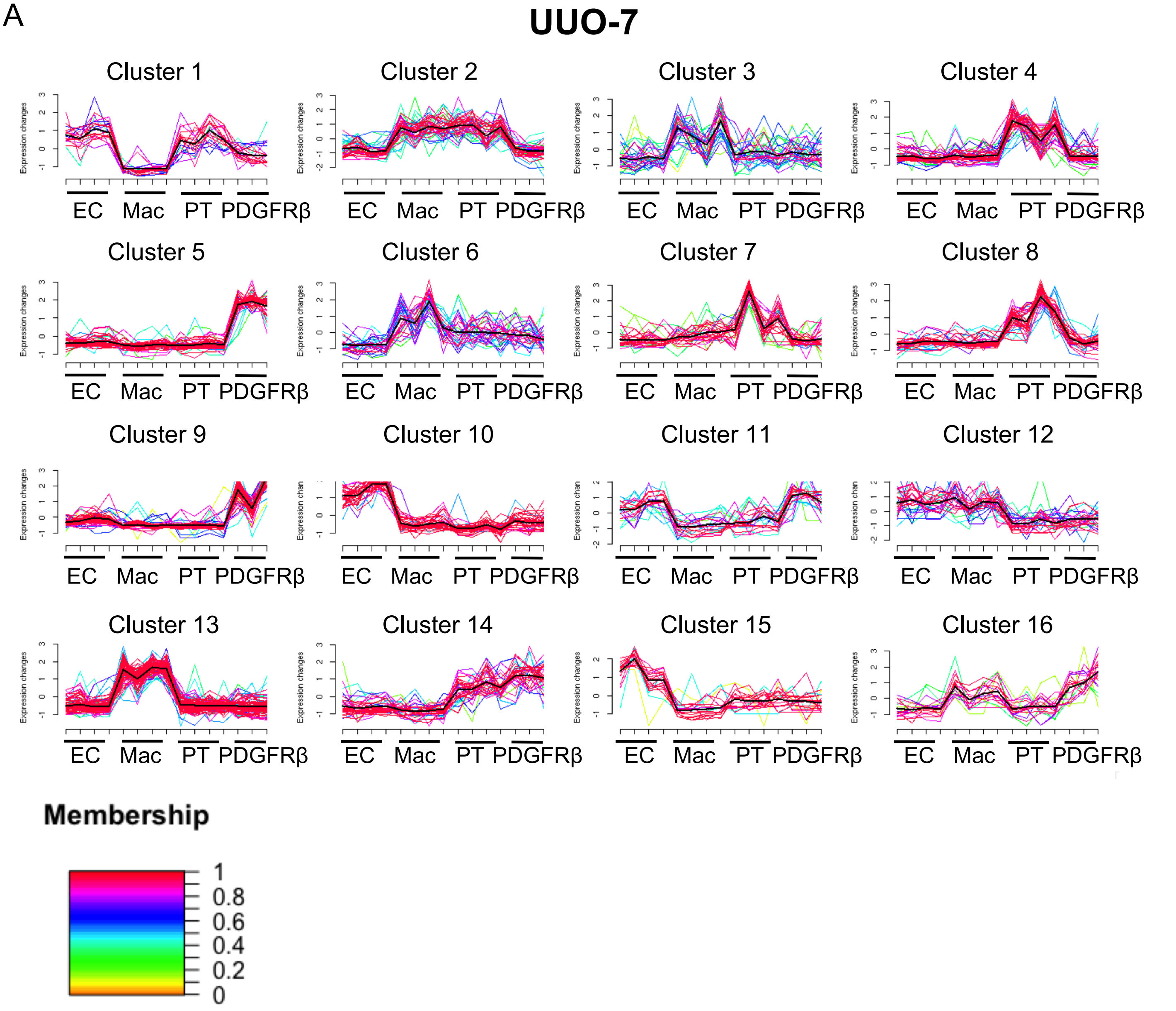

B

Cell Enrichment	UUO-7 mFuzz Cluster(s)
Macrophage (F4/80^{h})	3, 6, 13
Proximal Tubular (LTL^{+})	4, 7, 8
Fibroblast (PDGFRβ^{+})	5, 9
Endothelial (CD31^{+} CD45^{-})	10, 15
Not enriched by a single population	1, 2, 11, 12, 14, 16

Supplementary Figure 4: Mfuzz clustering of the single population data from UUO-7 timepoint. A: Clustering of single population sRNA-seq microRNAs by mFuzz demonstrates cell specific clusters (expression changes >0 in one cell type for >2 samples) for each of the isolated populations. The membership value colour indicates how well an individual expression pattern in an MFuzz cluster fits the dominant pattern in that cluster. Each downward tick on the x-axis represents a sample (n=4 for all cell types except PDGFRb (n=3)). **B:** Assignment of an mFuzz cluster to an enriched profile. mFuzz Parameters: Clusters 16, membership score 1.18.

A

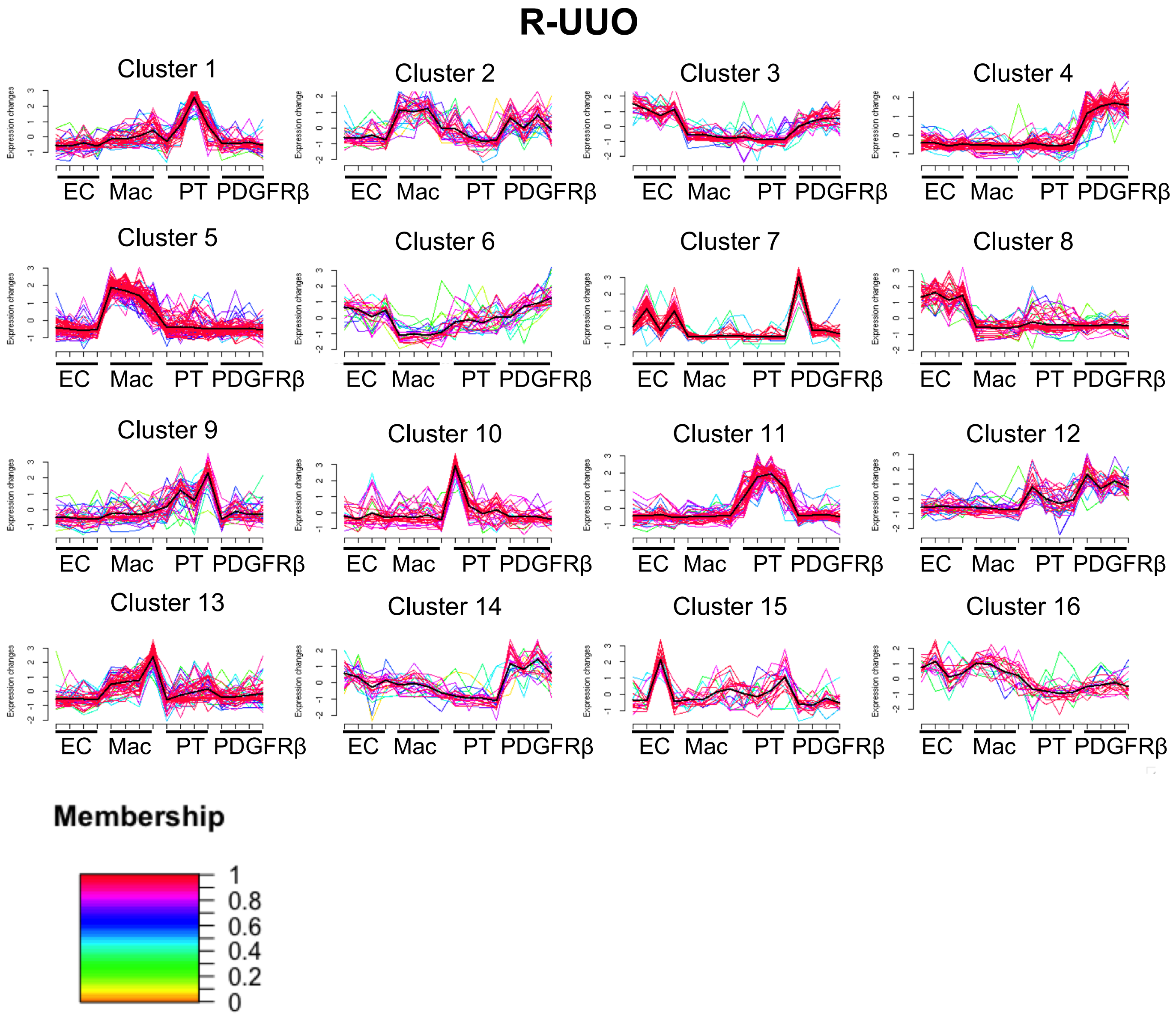

B

Cell Enrichment	R-UUO mFuzz Cluster(s)
Macrophage (F4/80^{hi})	5, 13
Proximal Tubular (LTL^{+})	1, 9, 11
Fibroblast (PDGFRβ^{+})	4, 14
Endothelial (CD31^{+} CD45^{-})	8
Not enriched by a single population	2, 3, 6, 7, 10, 12, 15, 16

Supplementary Figure 5: Mfuzz clustering of the single population data from the R-UUO timepoint. **A:** Clustering of single population sRNA-seq microRNAs by mFuzz demonstrates cell specific clusters (expression changes >0 in one cell type for >2 samples) for each of the isolated populations. The membership value colour indicates how well an individual expression pattern in an MFuzz cluster fits the dominant pattern in that cluster. Each downward tick on the x-axis represents a sample (n=4 for all cell types). **B:** Assignment of an mFuzz cluster to an enriched profile. mFuzz Parameters: Clusters 16, membership score 1.18.

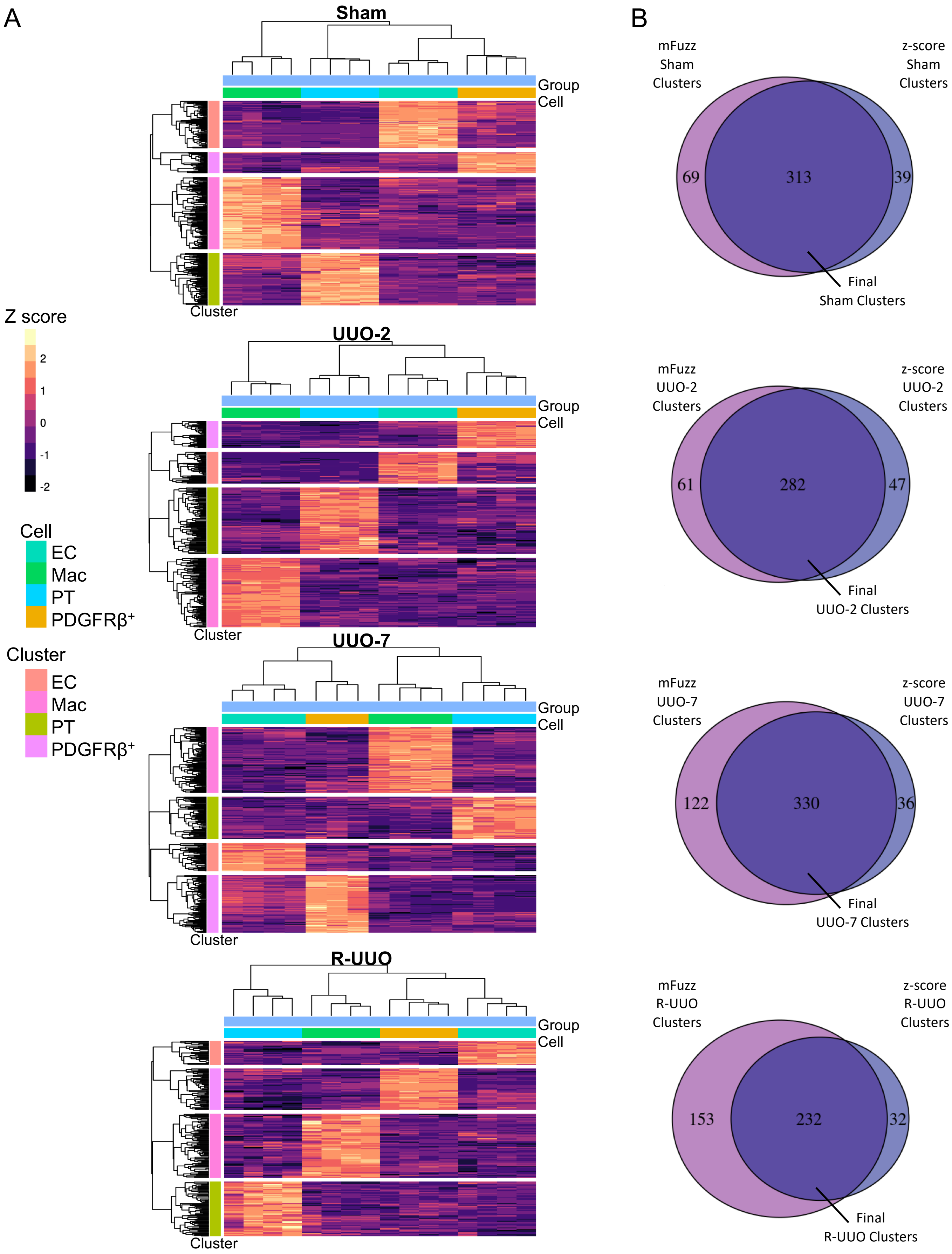

Supplementary Figure 6: Cell enriched microRNAs for each time point. A: Cell enriched microRNAs were first identified by selecting microRNAs with a higher expression (mean z-score of 1.15) relative to other cell types. As shown, at this level of stringency, the allocated cell 'profiles' of the microRNAs cluster to their cellular origin during unsupervised hierarchical clustering. **B:** The clusters identified by this method showed good overlap with those identified by mFuzz clustering. Those that were enriched by both methods were taken forwards as the final enriched clusters for each time point. In total, 432 unique microRNAs demonstrated some degree of enrichment using this approach. Each row represents a unique microRNA name and each column a sample from the single population sRNA-sequencing experiment.

A

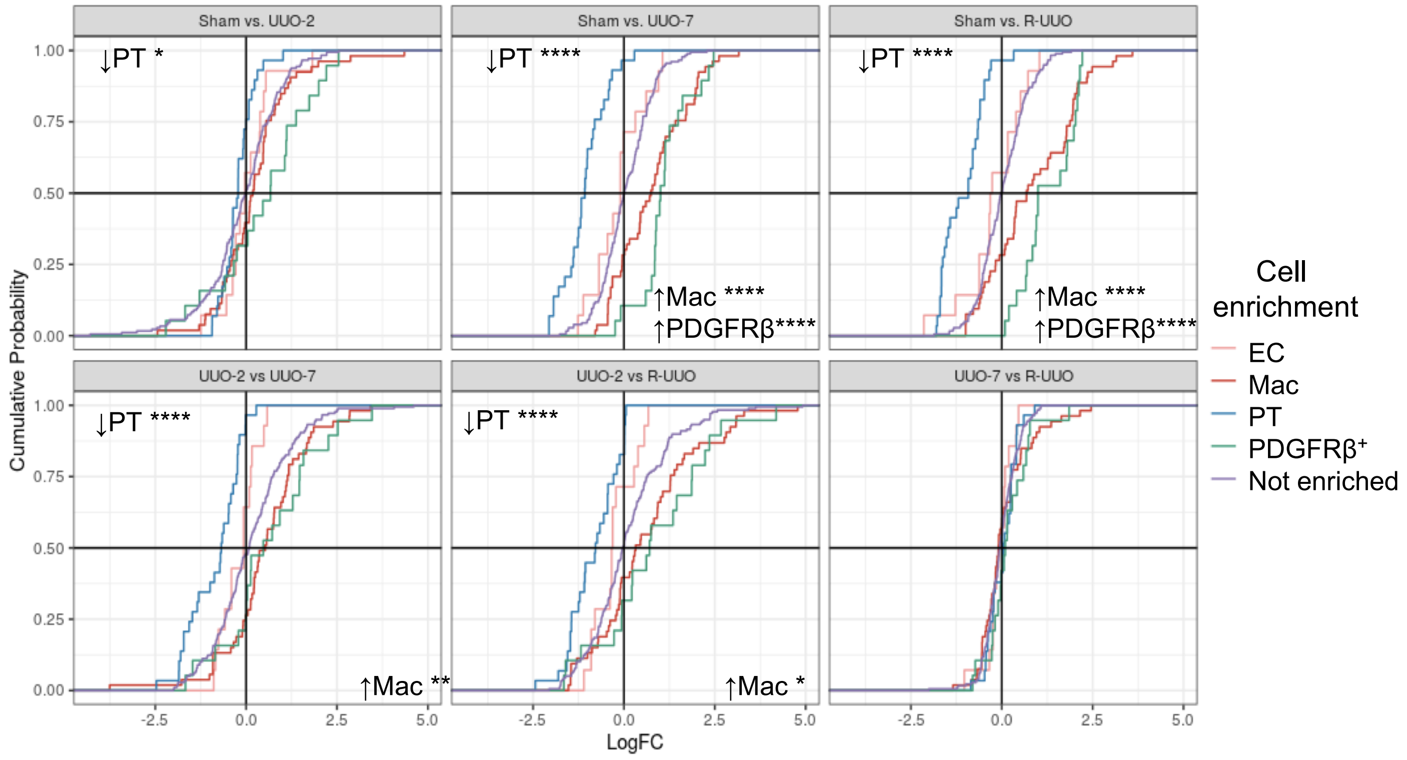

B

Comparison (vs. Non-Enriched miRs)	Macrophages	Proximal Tubular Cells	PDGFRb+ Cells	Endothelial Cells
Sham vs UUO-2	D= 0.156<br>p=0.268	D=0.299<br>**p=0.0281 ***	D=0.304<br>p=0.0843	D=0.220<br>p=0.5521
Sham vs UUO-7	D=0.349<br>**p<0.0001 ********	D=0.646<br>**p<0.0001 ********	D=0.679<br>**p<0.0001 ********	D=0.22<br>p=0.5568
Sham vs. R-UUO	D=0.377<br>**p<0.0001 ********	D=0.681<br>**p<0.0001 ********	D=0.690<br>**p<0.0001 ********	D=0.22<br>p=0.5711
UUO-2 vs. UUO-7	D=0.4879<br>**p=0.006****	D=0.448<br>**p<0.0001 ********	D=0.267<br>p=0.1729	D=0.298<br>p=0.1976
UUO-2 vs. R-UUO	D=0.21<br>**p=0.0429***	D=0.488<br>**p<0.0001 ********	D=0.326<br>**p=0.05187**	D=0.315<br>p=0.1531
UUO-7 vs. R-UUO	D=0.106<br>p=0.7845	D=0.166<br>p=0.5007	D=0.207<br>p=0.4557	D=0.1886<br>p=0.7451

Supplementary Figure 7: Expression of cell enriched microRNAs in the bulk sRNA-Seq. A: The cumulative distribution of the highly enriched microRNA expression changes (LogFC) in the bulk sRNA-sequencing for all comparisons are shown. **B**: Analysis of ECDF plots by Two-sample Kolmogorov-Smirnov test. *p<0.05, **p<0.01, ***p<0.001, ****p<0.0001.

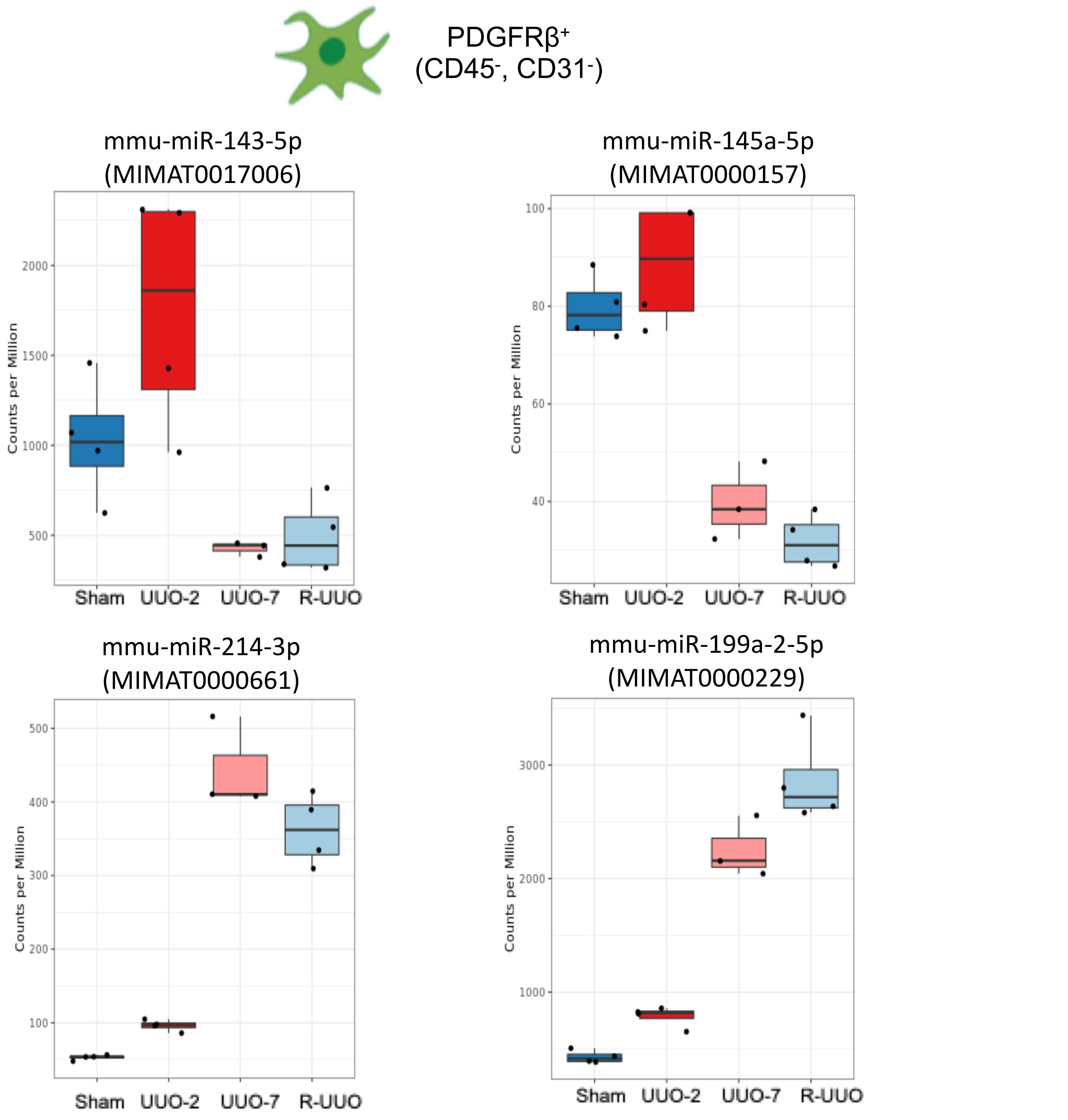

Supplementary Figure 8: Divergent expression of specific PDGFRβ+ cell enriched microRNAs. Within the PDGFRβ+ cells there is an increase in expression of the miR-199a/214 cluster and a decrease in expression miR143/145a cluster expression. Data from the http://www.kidney-enriched-micrornas.com/ interactive platform.

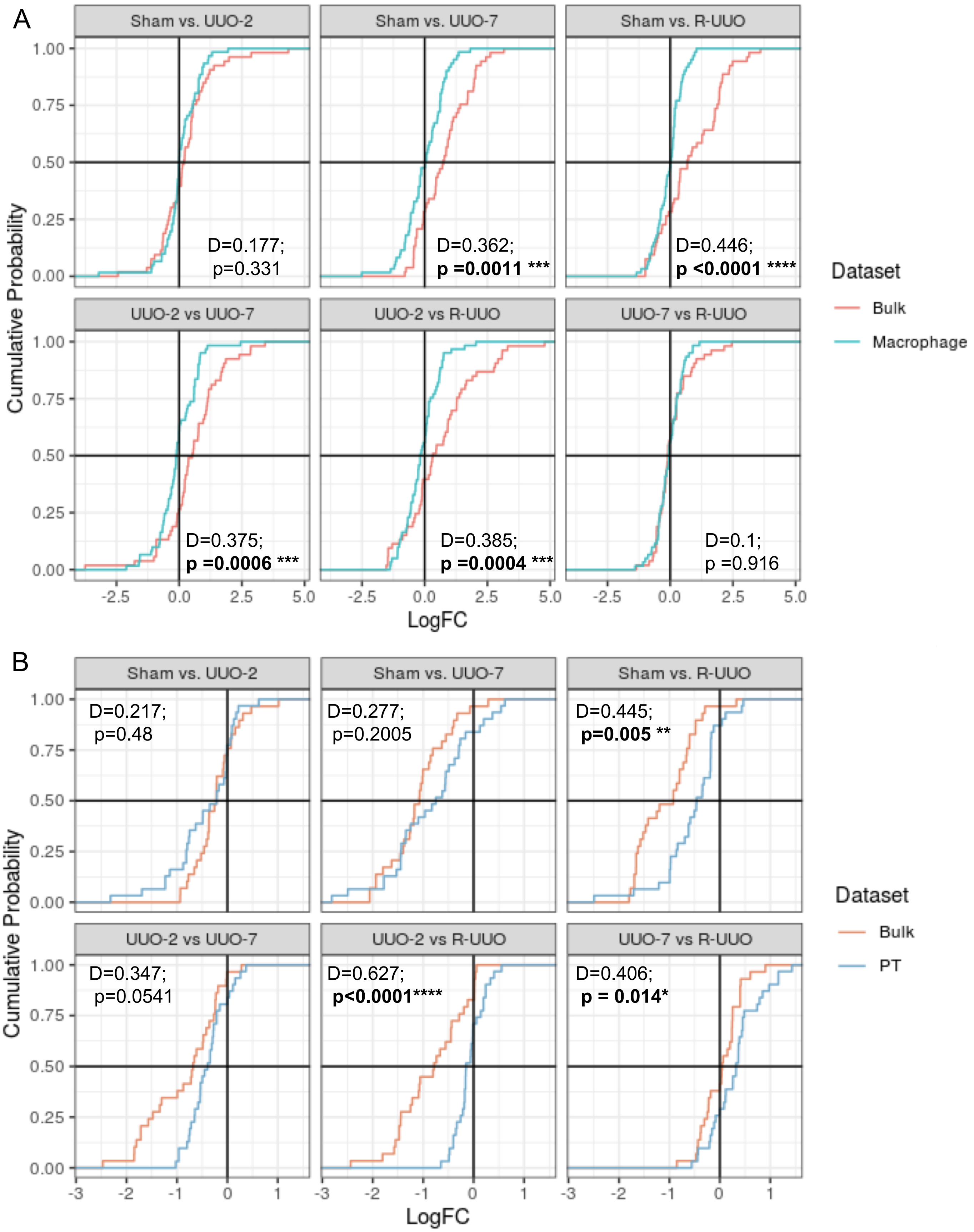

Supplementary Figure 9: Expression of cell enriched microRNAs in the bulk and single population sRNA-Seq with injury – Macrophage and Proximal Tubular Analysis A-B: Estimated Cumulative Distribution Frequency (ECDF) plots of the log fold changes for each injury comparison for enriched microRNAs in bulk vs. single population sRNA-seq datasets for Macrophages (A) and Proximal Tubular Cells (B). Analysis of ECDF plots by Two-sample Kolmogorov-Smirnov test. *p<0.05, **p<0.01, ***p<0.001, ****p<0.0001.

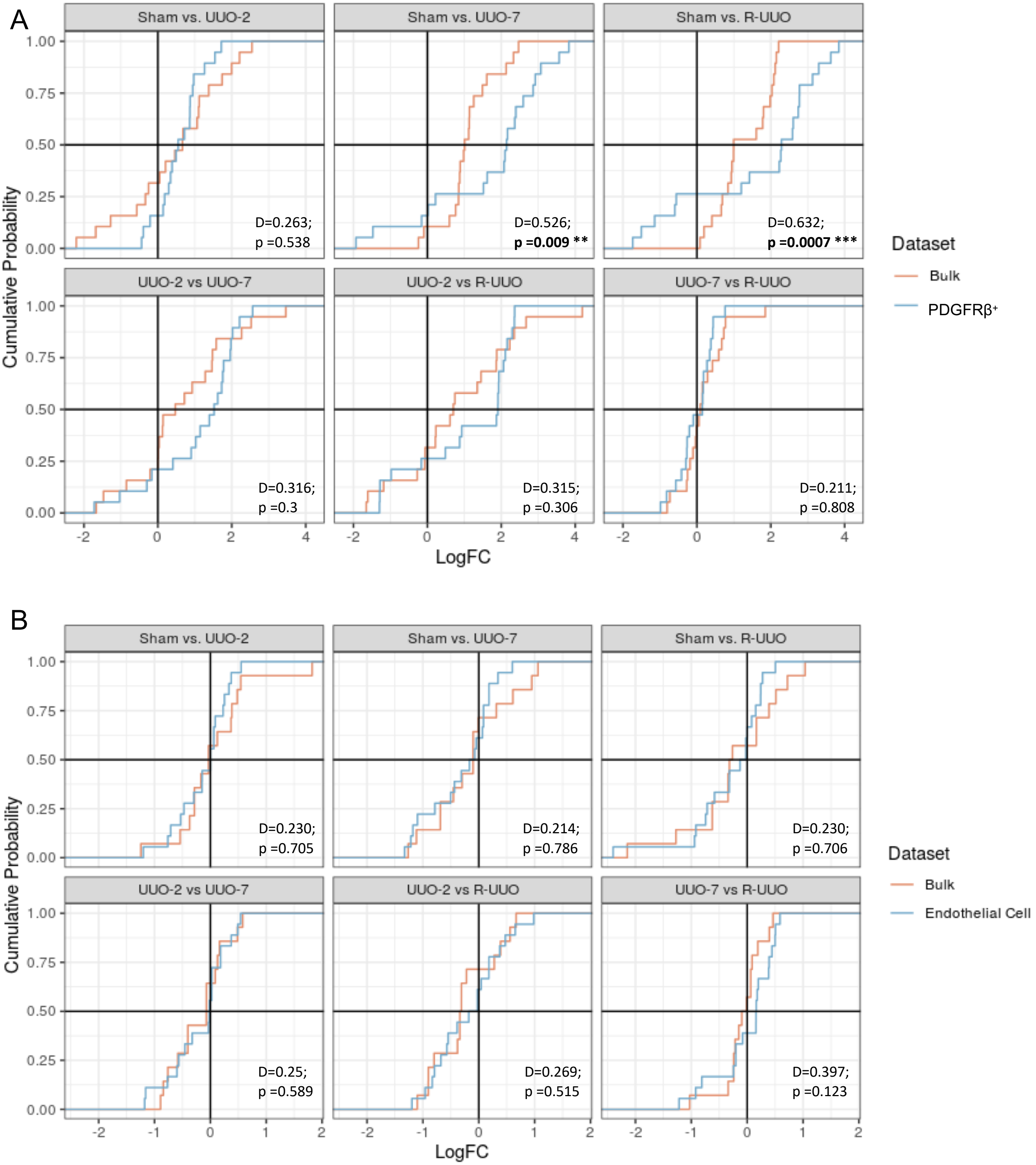

Supplementary Figure 10: Expression of cell enriched microRNAs in the bulk and single population sRNA-Seq with injury – PDGFRβ+ and Endothelial Cell Analysis A-B: Estimated Cumulative Distribution Frequency (ECDF) plots of the log fold changes for each injury comparison for enriched microRNAs in bulk vs. single population sRNA-seq datasets for PDGFRβ+ (A) and Endothelial Cells (B). Analysis of ECDF plots by Two-sample Kolmogorov-Smirnov test. *p<0.05, **p<0.01, ***p<0.001, ****p<0.0001.

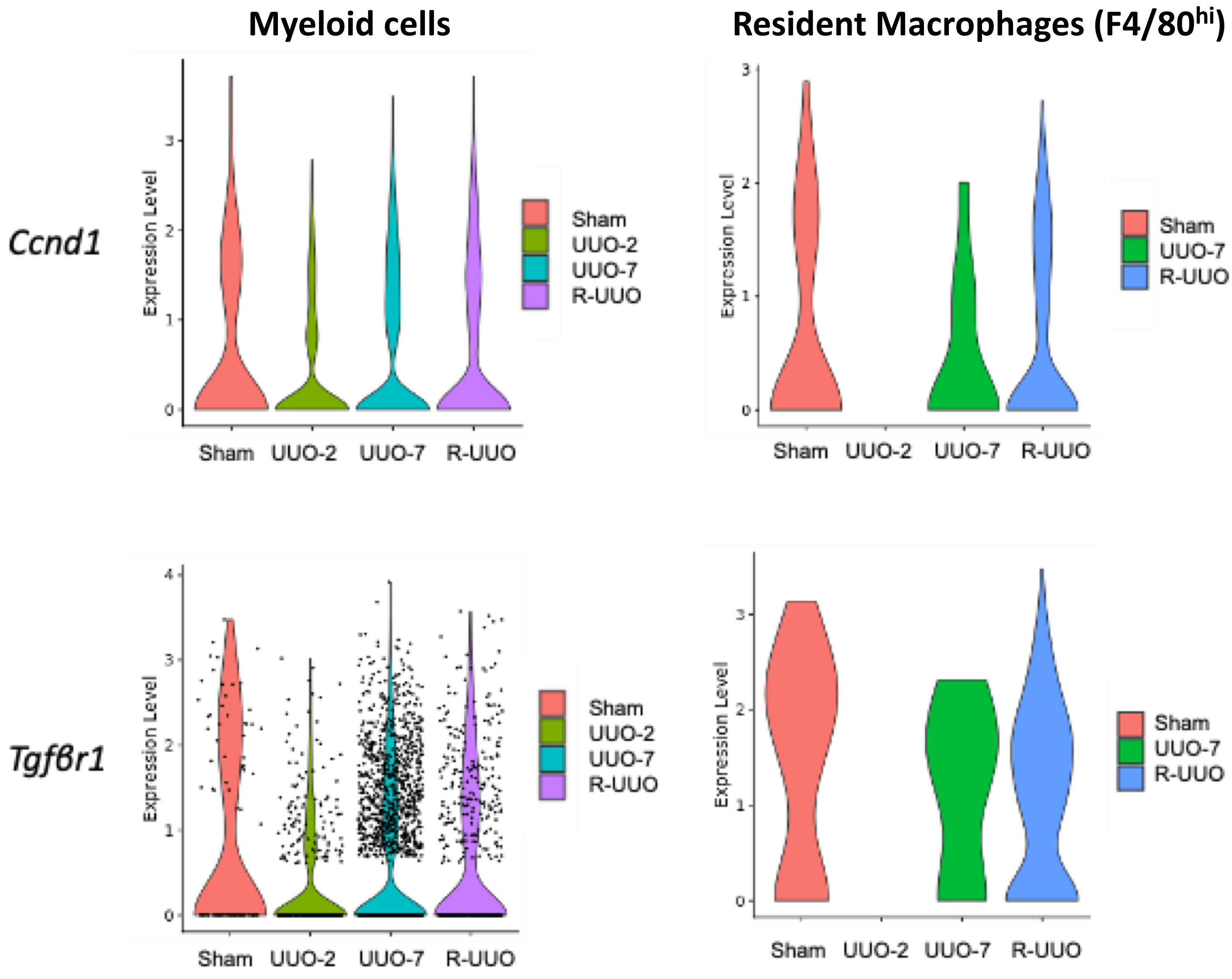

Supplementary Figure 11: Inverse expression of validated miR-18a-5p targets in the R-UUO kidney. Cross referencing the validated targets from Supplementary Table 1 with scRNA-seq from R-UUO published in Conway et al, 2020 reveals inverse expression of validated target genes of miR-18a-5p.

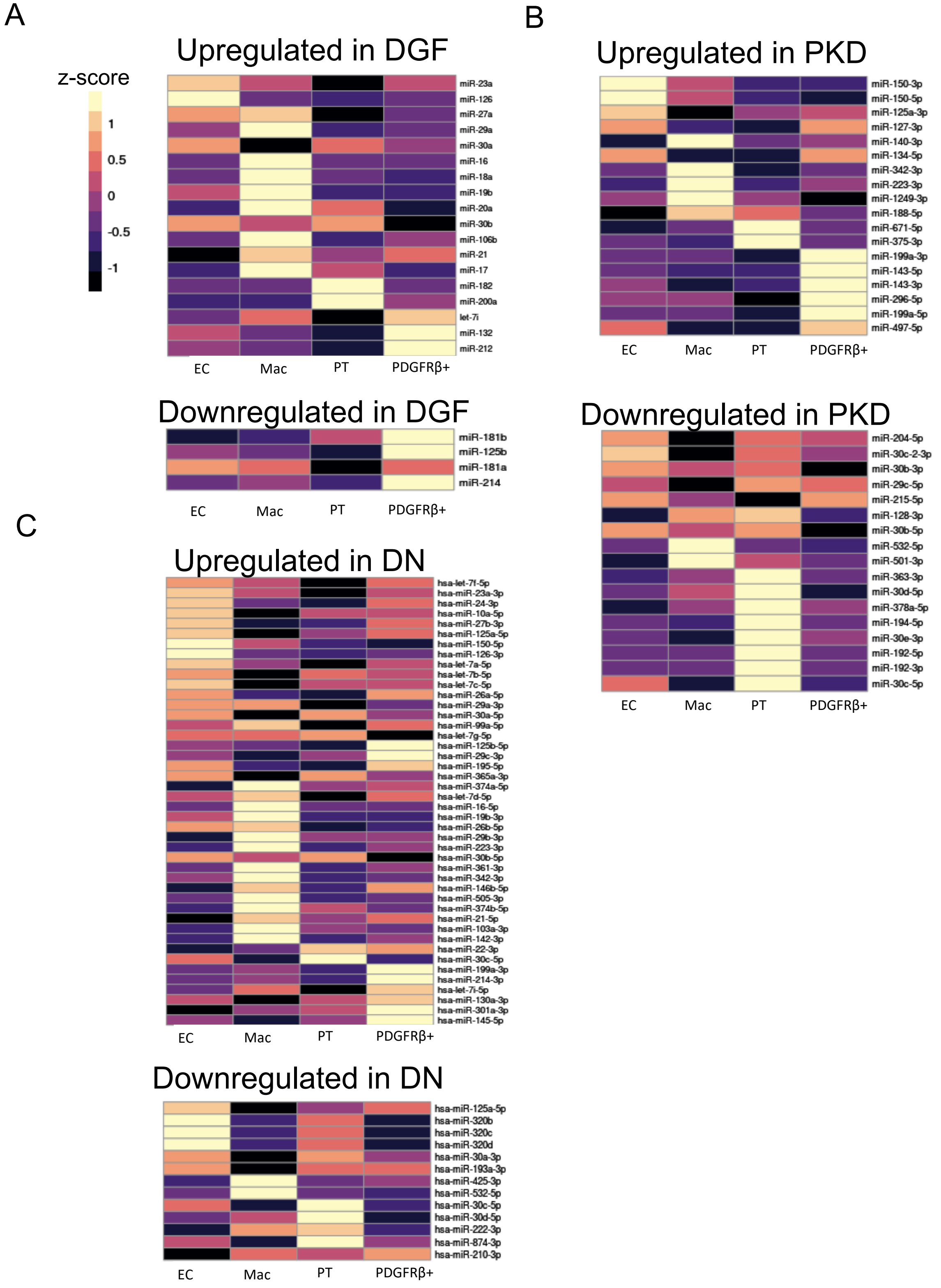

Supplementary Figure 12: Cell specificity of differentially expressed miRNAs in discrete renal disease phenotypes – microRNAs with any degree of enrichment are shown here. A-C: Cell population specific average expression of microRNAs reported to be differentially expressed in kidney biopsies of patients with delayed graft function, Diabetic Nephropathy (DN) and Polycystic Kidney Disease (PKD). Mean expression values of the microRNAs were calculated in each cell population and the colour scheme is based on the z-score distribution with microRNAs with all z-scores shown here. Macrophage (Mac), Endothelial Cells (EC), and Proximal Tubules (PT).

Author (Year)	Condition	Method	Source
Wilflingseder et. al. (2013)<br>Wilflingseder et. al. (2014)	Delayed Graft Function / Acute Tubular Necrosis	Microarray<br>Microarray	GEO2R data<br>GEO2R data
Kim DY et.al (2019)	APKD<br>Human (APKD renal cyst vs. Nephrectomy controls)	Microarray	Paper Supplementary data
Conserva et. al. (2019)	Diabetic Nephropathy (vs. Normal Kidney)	Microarray	Paper supplementary data

Supplementary Table 2: External data sources used to identify reported differentially expressed microRNAs in Figure 7 and Supplementary Figure 10. GEO2R: Gene Expression Omnibus Database.

Comparison	Sham vs. UUO Day 2	Sham vs. UUO Day 7	Sham vs. R-UUO	UUO Day 2 vs UUO Day 7	UUO Day 2 vs R-UUO	UUO Day 7 vs R-UUO
Early Injury Up	↑	−/↓	↑/−/↓	−/↓	−/↓	−/↓
Late Injury Up	−/↓	↑	↑/−/↓	−/↑	↑/−/↓	−/↓
Sustained Injury Up	↑	↑	↑/−/↓	↑/−/↓	−/↓	−/↓
Early Injury Down	↓	−/↑	↑/−/↓	−/↑	−/↑	−/↑
Late Injury Down	−/↑	↓	↑/−/↓	−/↓	−/↑	−/↑
Sustained Injury Down	↓	↓	↑/−/↓	↑/−/↓	−/↑	−/↑
Reversal Up	−/↓	−/↓	↑/−/↓	−/↓	↑in at least one comparison (cannot be ↓ in the other)	
Reversal Down	−/↑	−/↑	↑/−/↓	−/↑	↓ in at least one comparison (cannot be ↑ in the other)	
Any Injury Up, Reversal Down	↑in at least one comparison		↑/−/↓	↑/−/↓	↓ in at least one comparison	
Any Injury Down, Reversal Up	↓ in at least one comparison		↑/−/↓	↑/−/↓	↑in at least one comparison	

Supplementary Table 3: Logic arguments used to profile microRNAs with specific responses to injury and repair. ↑: Significantly up, ↓: Significantly down, − No significant change. Significance = False Discovery Rate < 0.05 and Fold Change > 1.5
